# Human papillomavirus (HPV) seroprevalence, cervical HPV prevalence and cervical lesions in systemic lupus erythematosus (SLE) and immunocompetent women

**DOI:** 10.1136/lupus-2025-001812

**Published:** 2026-04-09

**Authors:** Vanessa Infante, Karina Takesaki Miyaji, Camila Cristina Rodrigues Martini, Camila Picone, Sandra G Pasoto, Jose Eduardo Levi, Ana Carolina Soares Oliveira, Amanda Lara, Hanna Kann, Carina Eklund, Joakim Dilner, Cristina Paula Castanheira, Maricy Tacla, Paula Sato, Eloisa Bonfá, Philippe Mayaud, Ana Mari Christovam Sartori

**Affiliations:** 1 Departamento de Infectologia e Medicina Tropical, Universidade de Sao Paulo, São Paulo, Brazil; 2 Centro de Referencia para Imunobiologicos Especiais, Hospital das Clínicas da Faculdade de Medicina da Universidade de São Paulo, São Paulo, Brazil; 3 Departamento de Infectologia e Medicina Tropical, Universidade de Sao Paulo (FMUSP), São Paulo, Brazil; 4 Rheumatology, Universidade de Sao Paulo Faculdade de Medicina, São Paulo, Brazil; 5 Laboratorio de Investigacao Medica - Virologia (LIM-52), Hospital das Clínicas da Faculdade de Medicina da Universidade de São Paulo, São Paulo, Brazil; 6 Center for Cervical Cancer Elimination, Karolinska Institutet, Stockholm, Sweden; 7 Clinica de Ginecologia, FMUSP, São Paulo, Brazil; 8 Laboratorio de Investigacao Medica – Imunologia (LIM-48), Hospital das Clínicas da Faculdade de Medicina da Universidade de São Paulo, São Paulo, Brazil; 9 Faculty of Infectious and Tropical Diseases, London School of Hygiene & Tropical Medicine, London, UK

**Keywords:** Prevalence, Lupus Erythematosus, Systemic, Vaccination

## Abstract

**Introduction:**

Women with systemic lupus erythematosus (SLE) have increased prevalence of precancer cervical squamous intraepithelial lesions (SIL). This study aimed to compare human papillomavirus (HPV) seroprevalence, cervical HPV-DNA detection and HPV-related cervical lesions between SLE and immunocompetent women.

**Methods:**

In this cross-sectional study, women aged 18–45 years with SLE receiving immunosuppressive or disease-modifying antirheumatic drugs were compared with immunocompetent women, selected from the same hospital in Sao Paulo, Brazil. Eligible participants had no self-reported history of genital warts or any other HPV-related cervical, vaginal or vulvar lesions, nor received prior HPV vaccination. Cervical samples were tested for HPV-DNA using PapilloCheck and for SIL lesions by liquid-based cytology. A multiplex pseudovirion-based serology assay (PsV-Luminex) measured serum antibodies against eight of the HPV types targeted by non-valent (9vHPV) HPV vaccine.

**Results:**

A total of 122 women with SLE and 132 immunocompetent women (mean age 34.3 and 32.5 years, respectively) were enrolled. Compared with immunocompetent women, SLE participants had higher prevalence of HPV-related cervical lesions (low-grade and high-grade SIL combined, 11.3% vs 2.4%, p=0.009), higher prevalence of at least one cervical HPV type (38.8% vs 20.2%, p=0.003), non-significantly higher prevalence of cervical high-risk HPV types (23.5% vs 14.9% p=0.286) and higher HPV seroprevalence to eight HPV types included in the 9vHPV vaccine (80.4% vs 59.7%, p=0.001).

**Conclusion:**

Our findings underscore the importance of tailored cervical cancer screening and HPV vaccination of SLE women at young age.

**WHAT IS ALREADY KNOWN ON THIS TOPIC**

- Previous studies have demonstrated that women with systemic lupus erythematosus (SLE) have an increased risk of human papillomavirus (HPV) persistent infection, cervical cancer and precancerous lesions. Despite that, most current guidelines bring no specific cervical cancer screening recommendations for women with SLE and HPV vaccination coverage remains suboptimal in this population.

**WHAT THIS STUDY ADDS**

- This cross-sectional study integrated both molecular and serologic evidence of HPV exposure, providing novel insights into cumulative HPV exposure in immunocompromised women with SLE. The results evidenced higher HPV seroprevalence, higher cervical HPV-DNA positivity and higher prevalence of precancerous cervical lesions, in women aged 18–45 years with SLE receiving disease-modifying antirheumatic drugs or immunosuppressive therapy, in comparison to immunocompetent women of the same age.

**HOW THIS STUDY MIGHT AFFECT RESEARCH, PRACTICE OR POLICY**

- The findings support the importance of strengthening adherence to SLE-specific screening guidelines, expanding coverage of adolescent HPV vaccination and implementing targeted multiage catch-up strategies for unvaccinated high-risk immunocompromised women to decrease the burden of HPV disease in this population. HPV vaccines’ safety, immunogenicity and efficacy/effectiveness in immunocompromised need to be further evaluated.

## Introduction

 Human papillomavirus (HPV) infection is a leading cause of cancer globally. The International Agency for Research on Cancer has provided strong evidence linking HPV to cancers of the cervix, penis, vulva, vagina, anus and oropharynx.^1^ Cervical cancer is an advanced outcome of a persistent infection by high-risk HPV types (HR-HPV), defined by the detection of HPV-DNA in consecutive cervical specimen tests.^1^,^3^ HR-HPV 16 and 18 are responsible for about 70% of cervical cancers, and types 31, 33, 45, 52 and 58 together are responsible for about 20%.^1 2^

Cervical cancer is the fourth most common cancer in women globally with around 660 000 new cases and 350 000 deaths in 2022.^4^ Most deaths occur in low and middle-income countries.^4^ In Brazil, it reaches the third position, with 17 010 new cases estimated to occur in each year of the 2023–2025 triennium, corresponding to a crude incidence rate of 15.38 cases per 100 000 women, with regional differences.^5^

Women with systemic lupus erythematosus (SLE) have increased prevalence of precancerous squamous intraepithelial lesions (SIL) as compared with healthy women.^6 7^ A Brazilian study observed a high prevalence of malignant tumours in SLE women, particularly in those with longer disease duration. Breast and cervical cancer were the most frequent, both occurring at higher rates than in the general population.^8^

A nationwide Danish cohort of women with SLE, diagnosed from 1996 to 2021 and followed up in national registries for HPV-associated precancer and cancer up to 2022, found increased rates of cervical, vaginal, vulvar and anal precancers in SLE women compared with general population. SLE women had twofold higher risk of cervical precancer and up to fivefold increased risk of non-cervical anogenital precancers.^9^ Similarly, Australian population-based data also showed SLE women had higher rates of high-grade histological and cytological abnormalities compared with immunocompetent controls.^10^

The American College of Obstetricians and Gynecologists and the United States Preventive Services Task Force current guidelines bring no specific cervical cancer screening recommendations for women with SLE, differently from women living with HIV.^11 12^ In 2022, the Brazilian Ministry of Health published an SLE clinical protocol and therapeutic guideline, recommending annual gynaecological evaluation, including cervical tests to detect HPV-related cervical lesions and breast exams.^13^

This study aimed to assess HPV seroprevalence, cervical HPV-DNA prevalence and the frequency of HPV-related cytological lesions among SLE women in comparison to immunocompetent women of the same age.

## Methods

### Study populations and specimen collection

This cross-sectional study was conducted at the Reference Center for Special Immunobiologicals (*Centro de Referência para Imunobiológicos Especiais*, *CRIE*) of the Hospital das Clinicas, a tertiary/quaternary hospital attached to the Medical School of Sao Paulo University (HC-FMUSP), from 12 July 2017 to 16 October 2018.

Women aged 18–45 years with SLE (according to the 2012 classification criteria of Systemic Lupus International Collaborating Clinics)^14^ were enrolled at the Rheumatology Outpatient Clinic and CRIE of HC-FMUSP. Immunocompetent women of the same age, from the hospital staff, were included as comparator group. Inclusion criteria for SLE women were the use of disease-modifying antirheumatic drugs (DMARDs) or immunosuppressive therapy. For immunocompetent women, the inclusion criteria were absence of immunocompromise due to drugs or disease. Exclusion criteria for both groups were pregnancy or breastfeeding; any other causes of immunocompromise, such as HIV/AIDS, cancer or inborn errors of immunity; any biological therapy; heart, lung, liver, kidney or neurologic severe disease; self-reported history of genital warts or any HPV-related cervical, vaginal or vulvar lesions; and prior HPV vaccination. Rapid HIV and pregnancy tests were performed for all candidates at enrolment.

The following data were collected: age, ethnicity, educational level, age at menarche, age at sexual debut, number of sexual partners and previous sexually transmitted infections (STI) and known comorbidities. For SLE women, an index was used to evaluate the activity of disease activity (SLEDAI), defined according to the SLE Disease Activity Index 2000 score,^15^ and a cut-off SLEDAI-2K>4 was arbitrarily established as active disease. Data on DMARDs or immunosuppressive drugs and therapeutic regimens used were also collected. Body mass index was measured for all women. A 5 mL venous blood sample was collected for HPV serology. For women reporting having initiated sexual life, a speculum examination was performed by a trained nurse or physician during which a cytobrush was used to sample the ecto/endocervix. The sample was placed into a liquid-based cytology (LBC) vial (BD SurePath, TriPath, Burlington, North Carolina) and transported at room temperature to the laboratory on the same day.

### Laboratory testing

The LBC samples were vortexed for 20 s and processed using the fully automated BD Totalys system. This system features a multiprocessor that integrates the preprocessing of cytology samples with molecular testing, utilising 9 mL of SurePath liquid medium to perform ‘cell enrichment’, which removes potential interferences and forms an epithelial cell pellet using the SlidePrep equipment. The preprocessed material for oncotic cytology was then transferred to the BD Totalys SlidePrep automated medium for slide preparation and staining.

Cytological analysis was conducted at the Fundacao Oncocentro de São Paulo laboratory, a public reference centre for cytopathology. All cases were initially reviewed by cytotechnicians, and any suspected abnormalities were further evaluated by experienced cytologists and anatomopathologists. Results were reported in accordance with the Bethesda System for reporting cervical cytology, which includes the following categories: negative for intraepithelial lesion or malignancy; atypical squamous cells of undetermined significance; atypical squamous cells that cannot exclude high-grade SIL; low-grade SIL (LSIL), encompassing mild dysplasia/CIN 1; high-grade SIL (HSIL), encompassing moderate and severe dysplasia, carcinoma in situ, CIN 2 and CIN 3; atypical glandular cells favouring neoplasia or endocervical adenocarcinoma in situ.^16^ Participants with precursor lesions identified during the study were referred to gynaecologists for treatment.

Cervical samples were also screened for HPV-DNA using the PapilloCheck system (Greiner Bio-One, UK), a PCR-based DNA microarray able to identify 26 HPV types: 12 HR-HPV (16, 18, 31, 33, 35, 39, 45, 51, 52, 56, 58 and 59) and 14 HPV types considered as low risk (LR-HPV) or with limited evidence for cervical cancer in humans (6, 11, 40, 41, 42, 43, 44/55, 53, 66, 68, 70, 73 and 82).^17^ The test does not distinguish between HPV 44 and 55 due to cross-reactivity. All tests were conducted following the manufacturer’s instructions at the Laboratory of Medical Investigation/Virology, HC-FMUSP.

A multiplexed heparin-pseudovirion serology assay (PsV-Luminex) was used to detect type-specific anti-HPV antibodies. This assay employed pseudovirions containing L1 and L2 capsid proteins from nine HPV types (6, 11, 16, 18, 31, 33, 45, 52 and 58) to detect type-specific anti-HPV antibodies, as previously described.^18^ Testing was performed at the Center for Cervical Cancer Elimination, Karolinska Institutet, Sweden. The blood samples were stored at room temperature for no more than 8 hours before centrifugation, after which the serum samples were aliquoted, stored and transported at −20°C. All samples were analysed in conjunction one single time. The study population seropositivity was defined by calculating cut-off values for each HPV type analysing the mean fluorescence intensity (MFI) in 100 serum samples of Brazilian children aged 2–10 years. The global HPV LabNet algorithm was applied: the cut-off was determined as the mean MFI of a negative control serum panel plus three SDs. If the calculated cut-off was below 400 MFI, a default value of 400 MFI was used.

### Sample size calculation and statistical analysis

This analysis was conducted at the baseline of a trial that evaluated the quadrivalent HPV vaccine (4vHPV) immunogenicity and safety in SLE and solid organ transplanted women.^19 20^ So, the sample size calculation was based on the comparison of HPV seroconversion rates for each HPV type included in 4vHPV vaccine in SLE and immunocompetent women. The comparator group was common for both immunosuppressed groups. Based on the seroconversion rates found in a previous study conducted among SLE women,^21^ considering power of 80%, precision of 5% and assuming 10% of losses to follow-up until final evaluation, a sample of 125 was estimated necessary to detect a hypothesised 10% difference of postvaccination seroconversion rates between the SLE and immunocompetent groups.

A database was created using REDCap V.9.8.5 (2020, Vanderbilt University, Nashville). Statistical analyses were conducted using R for Windows, V.3.6.1. Descriptive statistics were used to describe the characteristics of population and results. Categorical variables, including HPV seroprevalence, HPV-DNA cervical prevalence and cervical intraepithelial lesions prevalence, were compared between the two groups using the χ^2^ test or Fisher’s exact test, as appropriate. Logistic regression was used to assess the association of SLE with cervical cytological lesions (HSIL or LSIL vs benign cellular changes), cervical HPV DNA positivity (any HPV type) and HPV seropositivity (any HPV type), adjusting for age, educational level and number of sexual partners. Among women with SLE, logistic regression was used to evaluate the association of current use of mycophenolate mofetil (MMF) and disease activity (SLEDAI ≤4=remission/low disease activity or >4=active disease) with HPV outcomes (cervical lesions, cervical HPV-DNA positivity (any HPV type) and HPV seropositivity (any HPV type), adjusting for age, educational level and number of sexual partners. Point estimates and 95% CIs were reported. Statistical significance was set at p<0.05.

### Patient and public involvement

Patients were not involved in the design of the study.

## Results

A total of 125 women with SLE and 132 immunocompetent women aged 18–45 years were enrolled. Due to eligibility criteria violation, three SLE participants were excluded: two women were misdiagnosed as SLE and another one was on anti-TNF therapy. Table 1 summarises the demographic and clinical characteristics of both groups’ participants. Compared with immunocompetent women, SLE women were slightly older (mean 34.4 vs 32.5 years) and had a lower educational level (mean 11.2 vs 14.3 years of schooling). There were no statistically significant differences in the number of lifetime sexual partners, history of STI and smoking habits between the two groups. Comorbidities, particularly arterial hypertension, hypothyroidism and dyslipidaemia, were also more common in SLE women, as expected.

**Table 1:** Demographic characteristics of participants with systemic lupus erythematosus (SLE) and immunocompetent women included in this study

	SLE	95% CI	Immunocompetent	95% CI	P value
	n=122		n=132		
Age (years)					
Mean (SD)	34.4 (7)	33.2 to 35.7	32.5 (6.3)	31.4 to 33.6	0.0289
Median (minimum–maximum)	36 (19–45)		33 (18–46)		
Ethnicity, n (%)					
White	64 (52.5)	43.9 to 61.8	74 (56.1)	47.5 to 64.3	0.9592
Black	22 (18.0)	12.5 to 26.5	20 (15.1)	10 to 22.4	
Brown	32 (26.3)	19.7 to 35.6	32 (24.2)	17.7 to 32.3	
Asian	2 (1.6)	0.4 to 6.5	6 (4.5)	2 to 9.8	
Duration of schooling (years)					
Mean (SD)	11.2 (3.3)	10.6 to 11.8	14.3 (3)	13.8 to 14.8	<0.0001
Median (minimum–maximum)	11 (0–19)		15 (3–22)		
Smoking, n (%)					
Yes	2 (1.6)	0.4 to 6.4	8 (6.1)	3 to 11.7	0.07
Age at menarche (years)					
Mean (SD)	13 (1.8)	12.6 to 13.3	12.5 (1.6)	12.2 to 12.8	0.0249
Median (minimum–maximum)	13 (8–19)		12 (9–18)		
Age at sexual debut (years)					
Mean (SD)	18 (3.3)	17.4 to 18.6	18.5 (3.1)	18 to 19.1	0.2125
Median (minimum–maximum)	17 (13–28)		18 (12–30)		
Number of lifetime sexual partners (%)					
0	8 (6.6)	3.3 to 12.6	5 (3.8)	1.6 to 8.8	0.1059
1–2	52 (42.6)	34.1 to 51.6	43 (32.6)	25.1 to 41	
3–5	43 (35.2)	27.3 to 44.1	60 (45.5)	37.1 to 54	
>5	16 (13.1)	8.2 to 2	24 (18.2)	12.5 to 25.7	
Unknown	3 (2.5)	0.8 to 7.4	0	NA	
History of sexually transmitted infections (STI), n (%)					
Yes	7 (5.7)	2.7 to 11.6	4 (3)	1.1 to 7.8	0.2979
Body mass index (kg/m^2^)					
Mean (SD)	26.9 (5.9)	25.8 to 28	25.4 (4.8)	24.5 to 26.2	0.0201
Median (minimum–maximum)	26 (14–48)		25 (17–40)		
Comorbidities, n (%)					
Arterial hypertension	61 (50.0)	41.2 to 58.8	5 (3.8)	1.6 to 8.8	<0.0001
Diabetes	1 (0.8)	0.1 to 5.6	1 (0.8)	0.1 to 5.2	0.9602
Hypothyroidism	17 (13.9)	8.8 to 21.3	5 (3.8)	1.6 to 8.8	0.0045
Dyslipidaemia	11 (9.0)	5 to 15.6	1 (0.8)	0.1 to 5.2	0.0042
Other	51 (41.8)	32.6 to 49.9	29 (22.0)	15.7 to 29.9	0.0007
None	18 (14.8)	9.5 to 22.2	88 (66.7)	58.2 to 74.2	<0.0001

Table 2 shows key disease and treatment parameters of SLE participants. Mean illness duration since SLE diagnosis was about 10 years. About half of the participants (50.8%) had inactive or mild SLE activity (SLEDAI-2K ≤4). The most used drugs were hydroxychloroquine (86.9%) and prednisone (68%), and most participants were under a three-drug regimen for disease control (45.1%).

**Table 2:** Clinical characteristics of 122 women with systemic lupus erythematosus (SLE) included in this study

SLE characteristics	Total (%)	95% CI
SLE’s years up to enrolment		
Mean (SD)	9.7 (7.3)	8.3 to 11.1
Median (minimum-maximum)	8 (0–30)	
SLE Disease Activity Index 2000 Score (SLEDAI-2K)		
Remission/low disease activity (≤4)	62 (50.8)	41.9 to 59.6
Active disease (>4)	52 (42.6)	34.1 to 51.6
Unknown	8 (6.5)	32.6 to 12.5
Disease-modifying antirheumatic drugs (DMARDs) and immunosuppressive drugs used		
Chloroquine	106 (86.9)	78.8 to 91.3
Prednisone	83 (68.0)	58.6 to 75.2
Mycophenolate mofetil (MMF)	51 (41.8)	33.6 to 51.2
Azathioprine	27 (22.1)	15 to 29.8
Cyclophosphamide pulse<30 days	4 (3.3)	1.2 to 8.4
Leflunomide	3 (2.5)	0.8 to 7.3
Pulse therapy with methylprednisolone<30 days	2 (1.6)	0.4 to 6.3
Tacrolimus	2 (1.6)	0.4 to 6.3

Cervical samples were collected from 114 SLE and 127 immunocompetent participants who had reported sexual debut. The results of the liquid-based cervical cytology showed higher prevalence of HPV-related cervical lesions (low (LSIL) and high (HSIL) grade SIL combined) in SLE women compared with immunocompetent women (11.3% vs 2.4%, p=0.009) (table 3). In the logistic regression, after adjustment for age, educational level and number of sexual partners, women with SLE had a statistically significant higher odds of cervical cytological lesions (LSIL or HSIL) compared with immunocompetent women (adjusted OR=6.62; 95% CI 1.65 to 26.66; p=0.008) (online supplemental table 1). Among women with SLE (n=90), current use of MMF was independently associated with cervical lesions (HSIL or LSIL), after adjustment for age, educational level and number of sexual partners (adjusted OR=5.08; 95% CI 1.38 to 18.79; p=0.015) (online supplemental table 2). Disease activity, assessed by the SLEDAI score, was not statistically associated with cervical lesions (HSIL or LSIL), after adjustment for age, educational level and number of sexual partners (adjusted OR=1.13; 95% CI 0.42 to 3.05; p=0.81) (online supplemental table 3).

**Table 3:** Results of the liquid-based cervical cytology for participants with systemic lupus erythematosus (SLE) and immunocompetent women

Cytology	SLE*	95% CI	Immunocompetent*	95% CI	P value†
	(N=114)		(N=127)		
	n (%)		n (%)		
Normal cytology/benign cellular changes	81 (71)	62 to 78.7	108 (85.0)	77.7 to 90.3	**0.009**
ASC-US	16 (14)	8.7 to 21.5	8 (6.3)	3.2 to 12.1	
ASC-H	4 (3.5)	1.3 to 9	8 (6.3)	3.2 to 12.1	
LSIL	11 (9.6)	5.4 to 16.6	2 (1.6)	3.9 to 6.1	
HSIL	2 (1.7)	0.4 to 6.8	1 (0.8)	0.1 to 5.4	

* Cervical samples collected only for women who had initiated sexual activity.

† Statistical analysis used Chi-squared test to compare LSIL+HSIL versus normal cytology/benign cellular changes. ASC-US and ASC-H were not included in the analysis.

ASC-H, atypical squamous cells, cannot exclude; ASC-US, atypical squamous cells of undetermined significance; HSIL, high-grade squamous intraepithelial lesion; LSIL, low-grade squamous intraepithelial lesion.

The results of HPV-DNA PCR tests in cervical samples are presented in table 4. Compared with immunocompetent women, SLE participants had significantly higher prevalence of at least one HPV type in cervical samples (38.8% vs 20.2%, p=0.003); and higher although not statistically significant prevalence of HR-HPV types (23.5% vs 14.9%, p=0.286). In the logistic regression, after adjustment for age, educational level and number of sexual partners, women with SLE had statistically significant higher odds of cervical HPV infection compared with immunocompetent women (adjusted OR=2.36; 95% CI 1.17 to 4.77; p=0.017) (online supplemental table 4). Among women with SLE (n=92), current use of MMF was associated with higher odds of cervical HPV positivity, after adjustment for age, educational level and number of sexual partners (adjusted OR=2.43; 95% CI 1.01 to 5.86; p=0.048). Disease activity, measured by SLEDAI score, was not associated with cervical HPV-DNA positivity after adjustment for age, educational level and number of sexual partners (adjusted OR=1.22; 95% CI 0.61 to 2.44; p=0.57) (online supplemental table 3).

**Table 4:** Results of HPV-DNA (PCR) in cervical samples of participants with systemic lupus erythematosus (SLE) and immunocompetent women

	SLE	95% CI	Immunocompetent	95% CI	P value
	n (%)		n (%)		
**HPV-PCR in cervical sample**	n=98*		n=114*		
Negative	60 (61.2)	50.8 to 70.9	91 (79.8)	71.3 to 86.7	
Positive	38 (38.8)	29.1 to 49.1	23 (20.2)	13.2 to 28.7	0.003
≥1 high-risk HPV type (HR-HPV)†	23 (23.5)	15.5 to 33.1	17 (14.9)	8.9 to 22.8	0.286
≥1 low-risk HPV type (LR-HPV)‡	15 (15.3)	8.8 to 24	6 (5.3)	1.9 to 11.1	0.286
Types included in the 4vHPV vaccine (6, 11, 16, 18)	7 (7.1)	2.9-14.2	2 (1.8)	0.2-6.2	0.299
Types included in the 9vHPV vaccine (6, 11, 16, 18, 31, 33, 45, 52, 58)	15 (15.3)	8.8-24	5 (5.3)	1.4-9.9	0.153
**Detected HPV type**	**n=38**		**n=23**		
6	2 (5.3)		0 (0.0)		
16	5 (13.2)		2 (8.7)		
31	2 (5.3)		0 (0.0)		
35	2 (5.3)		0 (0.0)		
39	4 (10.5)		3 (13.0)		
40	2 (5.3)		0 (0.0)		
42	7 (18.4)		2 (8.7)		
45	3 (7.9)		1 (4.3)		
51	3 (7.9)		6 (26.1)		
52	2 (5.3)		2 (8.7)		
53	3 (7.9)		6 (26.1)		
56	3 (7.9)		3 (13.0)		
58	4 (10.5)		1 (4.3)		
59	1 (2.6)		3 (13.0)		
66	3 (7.9)		0 (0.0)		
68	2 (5.3)		1 (4.3)		
70	2 (5.3)		0 (0.0)		
73	2 (5.3)		0 (0.0)		
44/55	7 (18.4)		2 (8.7)		

* 30 cervical samples (17 from SLE women and 13 from immunocompetent women) were lost due to low quality.

† HR-HPV types tested: 16, 18, 31, 33, 35, 39, 45, 51, 52, 56, 58 and 59.

‡ LR-HPV types tested: 6, 11, 40, 41, 42, 43, 44/55, 53, 66, 68, 70, 73 and 82.

HPV, human papillomavirus; HR-HPV, high-risk HPV; LR-HPV, low-risk HPV.

HPV-DNA of types included in the 4vHPV vaccine was detected in 7.1% and 1.8% of SLE and immunocompetent participants, respectively (p=0.299), whereas HPV-DNA of eight types included in the 9vHPV vaccine was detected in 15.3% and 4.4% (p=0.153) (table 4).

HPV types most frequently detected in the SLE group were 42 and 44/55, both LR-HPV. In contrast, in the immunocompetent group, the most frequent HPV types were 51 (HR-HPV) and 53 (LR-HPV) (table 4).

HPV serology was done for 107 SLE women and 119 immunocompetent women. Seropositivity for at least one HPV type was significantly higher in the SLE group (80.4% vs 59.7%, p=0.001) (table 5). SLE participants also showed significantly higher seropositivity for at least one HR-HPV (80.4%) than immunocompetent (53.8%, p=0.003). In the logistic regression, after adjustment for age, educational level and number of sexual partners, women with SLE had statistically significant higher odds of HPV seropositivity compared with immunocompetent women (adjusted OR=2.58; 95% CI 1.28 to 5.20; p=0.008). The number of sexual partners was also independently associated with HPV seropositivity (adjusted OR per additional partner=1.21; 95% CI 1.06 to 1.37; p=0.003; online supplemental table 5). Among women with SLE (n=103), after adjustment for age, educational level and number of sexual partners, neither MMF use (adjusted OR=0.98; 95% CI 0.35 to 2.70; p=0.967) nor disease activity, assessed by the SLEDAI score (adjusted OR=1.09; 95% CI 0.47 to 2.55; p=0.84; online supplemental table 3), was associated with HPV seropositivity.

**Table 5:** Results of HPV serology (PseudoVirion-Luminex) for participants with systemic lupus erythematosus (SLE) and immunocompetent women

	SLE	95% CI	Immunocompetent	95% CI	P value
	n (%)		n (%)		
**HPV serology**	**n=107**		**n=119**		
Positive	86 (80.4)	71.7 to 86.9	71 (59.7)	50.6 to 68.1	0.001
Negative	21 (19.6)	13.1 to 28.3	48 (40.3)	31.9 to 49.4	
≥1 high-risk HPV type (HR-HPV)*	86 (80.4)	71.6 to 87.4	64 (53.8)	44.4 to 63	0.003
≥1 low-risk HPV type (LR-HPV)†	35 (32.7)	24 to 42.5	33 (27.7)	19.9 to 36.7	0.467
4vHPV vaccine types (6, 11, 16, 18)	77 (72.0)	62.4 to 80.2	59 (49.6)	40.2 to 58.9	0.001
8 of 9vHPV vaccine types‡	86 (80.4)	71.6 to 87.4	71 (59.7)	50.3 to 68.5	0.001
**Detected HPV type**	**n=86**		**n=71**		
6	29 (33.7)		28 (39.4)		
11	21 (24.4)		11 (15.5)		
16	59 (68.6)		32 (45.1)		
18	49 (57.0)		24 (33.8)		
31	20 (23.3)		13 (18.3)		
33	18 (20.9)		12 (16.9)		
52	25 (29.1)		23 (32.4)		
58	27 (31.4)		15 (21.1)		

* HR-HPV types tested:16, 18, 31, 33, 45, 52, 58; HPV-45 was not tested due to a technical issue.

† LR-HPV types tested 6, 11.

‡ 8 of the 9vHPV tested: 6, 11, 16, 18, 31, 33, 52, 58; HPV-45 was not tested due to a technical issue.

HPV, human papillomavirus.

Seropositivity to HPV types included in the 4vHPV (72% vs 49.6%, p=0.001) and 9vHPV vaccines (80.4% vs 59.7, p=0.001) was also higher in SLE participants compared with immunocompetent women.

## Discussion

This study demonstrates a consistent pattern of increased prevalence of HPV infection and HPV-related cervical lesions among women with SLE, underscoring the importance of tailored preventive strategies in this population. Compared with immunocompetent women, SLE participants had statistically significant higher rates of HPV cervical infection and HR-HPV types. Precancer lesions (HSIL and LSIL) were nearly five times more prevalent.

These findings are consistent with previous reports. A systematic review and meta-analysis^22^ showed higher prevalence of HPV in cervical samples in women with SLE (34.15%) compared with healthy women (15.3%).^22^ Another recently published review found a prevalence of cervical HPV infection around 25% (range, 14.7%–29%) among women with SLE, similar to that observed in women living with HIV, despite the heterogeneity between studies regarding country, design and number of participants.^23^ This review also found SLE women have higher frequency of multiple HR-HPV infections, which confer higher risk of cervical cancer.^23^ Additionally, a USA study involving 30 women with moderate/severe SLE on immunosuppressive therapy and with risk factors for HPV infection (multiple sexual partners, not using condoms, with history of abnormal pap smears and unvaccinated for HPV) reported HPV-DNA positivity in 70% of vaginal samples, with active infection (HPV-RNA positive) in all positive cases.^12^

Cervical cytological abnormalities are also more prevalent in SLE women even after controlling for sociodemographic and behavioural factors. This finding suggests that SLE, as a clinical condition, may contribute to an increased susceptibility to cervical epithelial abnormalities beyond traditional risk factors. The combined prevalence of high-grade and low-grade SIL in the SLE population (11.3%) is in line with prior Brazilian and Mexican studies, which reported abnormal Pap smear rates of 9.4% and 5.9%, respectively.^24 25^ In the recently published review, a meta-analysis pooling seven studies found an OR of 8.66 (95% CI 3.75 to 20.00) for the risk of high-grade cervical lesions in SLE women (n=416) versus controls (n=11 408).^23^ Some studies did not find a clear association between high-grade lesions and the type of immunosuppressive therapy,^21 22^ suggesting that the immune dysfunction inherent to SLE might play a more central role. Another Brazilian study did not observe higher frequency of colposcopy abnormalities in SLE women using immunosuppressants.^25^ However, the small number of HSIL cases limits more definitive conclusions. It is important to point out that all SLE women enrolled in our study were on DMARDs or immunosuppressive therapy, and so, we were not able to evaluate the effect of treatment, but current use of MMF was statistically associated with increased odds of cervical lesions (HSIL or LSIL) and cervical HPV infection. Other studies reported an association of HPV infection and long-term use of corticosteroids, methotrexate, azathioprine and cyclophosphamide.^23^ These findings support the hypothesis that immunosuppressive therapy may contribute to cervical lesions’ progression, possibly through impaired viral clearance.

A strength of our study was the integration of both molecular and serologic evidence of HPV exposure, with rigorous laboratory methods for HPV-DNA detection and HPV serology. To our knowledge, this is the first study to report seroprevalence of multiple HPV types in women with SLE, providing novel insights into cumulative HPV exposure in this population, and supporting the importance of both vaccination at an early age and screening strategies. PsV-Luminex serology provides a sensitive measure of prior exposure, but seropositivity does not distinguish between active and cleared infection. We found statistically significant higher seropositivity to at least one HPV type in the SLE group (80.4%) compared with immunocompetent women (59.7%), even after controlling for behavioural and sociodemographic factors. This finding suggests increased cumulative exposure and/or impaired immune-mediated viral clearance among women with SLE. Notably, significantly higher seroprevalence of HPV types included in the 4vHPV in SLE women (72.0%) than in immunocompetent (49.6%) was detected, whereas cervical HPV-DNA positivity for the 4vHPV vaccine types was much lower in both groups (7.1% and 1.8%, respectively), suggesting previous exposure and clearance. The seroprevalence of eight HPV types included in the 9vHPV vaccine was even higher (80.4% in SLE and 59.7% in immunocompetent participants). These findings reinforce the importance of HPV vaccination prior to sexual debut and suggest that 9vHPV may confer broader protection in this high-risk population, which needs further appropriate evaluation.

This study had several limitations. First, the study design (cross-sectional) limits causal inference, since temporality of SLE status and HPV outcomes is lacking. Furthermore, it does not allow to assess HPV persistence, which is critical to the progression to cervical cancer. Second, the sample size was powered to evaluate 4vHPV vaccine immunogenicity and may have been underpowered to detect differences in prevalence of HR-HPV or cytological lesions between groups. Third, the comparator group was constituted mainly by hospital staff, who may have socioeconomic and behavioural differences in comparison to the SLE group. This selection bias could explain, at least partly, differences in HPV exposure and seroprevalence. However, although women with SLE were a little older and less educated than the immunocompetent, there were no significant differences in the number of lifetime sexual partners, the history of STI and smoking. Furthermore, adjusting for age, educational level and number of partners has not changed the main results.

Beyond biological vulnerability, preventive care gaps may also contribute to increased cervical cancer risk in women with SLE. A study from the University of Washington showed that only 38% were up to date with their screening according to the American Society for Colposcopy and Cervical Pathology guidelines, pointing to a need for increased awareness and consensus among interdisciplinary providers regarding SLE-specific cervical cancer screening.^26^ In Brazil, the cervical cancer screening coverage among women with SLE is unknown. Among the general female population, cervical cancer screening coverage in the capital cities has remained close to 80% over the past decade. A decline was observed in 2020 and 2021, possibly as a result of the restrictions related to the COVID-19 pandemic.^5^

HPV vaccination coverage also remains suboptimal. A French study showed low HPV vaccination rates (<50%) among SLE and juvenile idiopathic arthritis adolescents. Limited knowledge, lack of recommendation by a health professional and fear of vaccine adverse events were the main barriers.^27^ In Brazil, HPV vaccination was introduced in 2014, only for girls at first. It became a gender-free programme in 2017, targeting girls and boys aged 9–14 years. The 4vHPV vaccine coverage has increased slowly and reached 82.6% among girls aged 9–14 years and 67% among boys of the same age, in 2024, with great regional differences.^28^ Since 2017, immunocompromised persons aged 9–45 years, including those with SLE, have been progressively included in the HPV vaccination programme, but 4vHPV vaccine coverage among Brazilian women with SLE is unknown.

Low coverage of screening programmes and restricted recommendations of HPV vaccination limit their potential for cancer elimination. HPV vaccination of HPV-naïve women aged up to 45–55 years has been shown to be safe, immunogenic and conferred protection of about 90% against cervical precancer lesions caused by HPV vaccine types.^29^,^31^ A combined protocol of catch-up HPV vaccination of women aged up to 45–50 years at the time of their HPV-screening test, with appropriate follow-up and treatment for women with positive test, has the potential to accelerate the decline of cervical cancer incidence and mortality.^32^ Such a strategy, called ‘HPV Faster’, proposed in 2016, is being introduced under an implementation project in some European countries.^33^

In conclusion, our findings underscore the need for disease-specific preventive care protocols for women with SLE. Strengthening adherence to SLE-specific screening guidelines, expanding coverage of adolescent vaccination and implementing targeted multiage catch-up strategies for unvaccinated high-risk immunocompromised women are essential measures. The potential added benefit of switching from quadrivalent to 9-valent HPV vaccine in this high-risk population should be further explored, since vaccines were not evaluated in this study.

## Supplementary material

10.1136/lupus-2025-001812 online supplemental file 1

10.1136/lupus-2025-001812 online supplemental file 2

10.1136/lupus-2025-001812 online supplemental file 3

## Data Availability

Data are available upon reasonable request.
