# Supplementary material for: Human papillomavirus (HPV) seroprevalence, cervical HPV prevalence and cervical lesions in systemic lupus erythematosus (SLE) and immunocompetent women
Source: Lupus Sci Med. 2026 Apr 9;13(1):e001812. doi: 10.1136/lupus-2025-001812 (PMC13084834; doi:10.1136/lupus-2025-001812)
Supplement: online supplemental file 1 [file lupus-13-1-s001.pdf]

Avaliação da resposta imune primária e segurança da vacina de HPV em mulheres em uso de drogas imunossupressoras devido a transplante de órgãos sólidos (rim ou fígado) ou doença reumatológica (Lúpus Eritematoso Sistêmico)

Participantes:
Ana Marli Christovam Sartori (Coordenadora)
Departamento de Moléstias Infecciosas e Parasitárias da Faculdade de Medicina da Universidade de São Paulo (FMUSP)
Curriculum lattes: http://lattes.cnpq.br/8455588138553589

Karina Takesaki Miyaji
Clínica de Moléstias Infecciosas e Parasitárias / Centro de Referência para Imunobiológicos Especiais (CRIE) do Hospital das Clínicas da FMUSP (HCFMUSP)
Curriculum lattes: http://lattes.cnpq.br/1088809698052835

Vanessa Infante
Clínica de Moléstias Infecciosas e Parasitárias / Centro de Referência para Imunobiológicos Especiais (CRIE) do HCFMUSP
Curriculum lattes: http://lattes.cnpq.br/8310264849350256

Maricy Tacla Alves Barbosa
Clinica de Obstetrícia e Ginecologia do HCFMUSP
Curriculum lattes: http://lattes.cnpq.br/4126651066831845

José Eduardo Levi
Instituto de Medicina Tropical da USP (IMT-USP)
Curriculum lattes: http://lattes.cnpq.br/6216061943429311

Philippe Mayaud
London School of Hygiene and Tropical Medicine (LSTMH), Inglaterra
Email: Philippe.Mayaud@lshtm.ac.uk

Joakim Dillner
Department of Laboratory Medicine, Karolinska Institute, Suécia
Email: joakim.Dillner@ki.se

William Carlos Nahas
Professor Titular de Urologia da FMUSP e Chefe do Serviço de Transplante Renal do HCFMUSP
Curriculum lattes: http://lattes.cnpq.br/3435741810254100

Luiz Augusto Carneiro D'Albuquerque
Professor Titular de Gastroenterologia da FMUSP e Chefe da Divisão de Transplante de Fígado e Órgãos do Aparelho Digestivo do HCFMUSP
Curriculum lattes: http://lattes.cnpq.br/3456781077547136

Eloisa Bonfá
Professora Titular de Reumatologia da FMUSP, Responsável pelo Ambulatório de Lupus do HCFMUSP
Curriculum lattes: http://lattes.cnpq.br/1810614032141202

Instituições participantes

Departamento de Moléstias Infecciosas e Parasitárias da Faculdade de Medicina da Universidade de São Paulo (FMUSP)

Clínica de Moléstias Infecciosas e Parasitárias do Hospital das Clínicas da Faculdade de Medicina da Universidade de São Paulo (HCFMUSP)

Centro de Referência para Imunobiológicos Especiais do Hospital das Clínicas da Faculdade de Medicina da Universidade de São Paulo (HCFMUSP)

Clínica de Ginecologia do Hospital das Clínicas da Faculdade de Medicina da Universidade de São Paulo (HCFMUSP)

Serviço de Reumatologia do Hospital das Clínicas da FMUSP

Divisão de Transplante de Fígado e Órgãos do Aparelho Digestivo do Hospital das Clínicas da FMUSP

Serviço de Transplante Renal do Hospital das Clínicas da FMUSP

Laboratório de Virologia Instituto de Medicina Tropical da USP (IMT-USP)

London School of Tropical Medicine and Hygiene (LSTMH)

Department of Laboratory Medicine, Karolinska Institute, Suécia

Sumário

Resumo 5
Abstract: 6
1. Enunciado do problema: 7
1.1 Papiloma Vírus Humano 7
1.2 Vacinas de HPV 8
2. Justificativa para realização do estudo: 12
3. Objetivos: 13
4. Métodos: 13
4.1 Desenho do estudo: 13
4.2 População do estudo: 14
4.2.1 Critérios de inclusão: 14
4.2.2 Critérios de exclusão 14
4.2.3 População fonte do estudo e área de recrutamento 15
4.3 Desfechos do estudo 15
4.3.1. Desfecho primário de imunogenicidade: 15
4.3.2. Desfecho primário de segurança: 16
4.3.3. Desfechos secundários: 19
4.4. Variáveis de interesse 20
4.5. Procedimentos para realização do estudo 20
4.6. Critérios de retirada do estudo 21
4.7. Metodologia laboratorial: 21
4.7.1. Detecção e dosagem de anticorpos contra HPV: 21
4.7.2. Detecção e genotipagem de HPV 22
4.7.3. Armazenamento do material biológico 22
4.8. Tamanho da amostra e poder estatístico 23
4.8.1. Cálculo da amostra 23
4.9. Análise estatística 25
4.9.1. Análise de imunogenicidade 25
4.9.2. Análise de segurança: 26
5. Questões éticas 27
6. Resultados Esperados 27
7. Desafios científicos e tecnológicos e os meios e métodos para superá-los: 28
8. Formação de recursos humanos 28
9. Disseminação e avaliação: 28
10. Orçamento 28
11. Financiamento 28
12. Outros apoios ao estudo: 28

14. Cronograma de realização do estudo* 31
15. Bibliografia 32

Resumo

Título do projeto de pesquisa: Imunogenicidade e segurança da vacina de HPV em mulheres em uso de drogas imunossupressoras devido a transplante de órgãos sólidos (rim ou fígado) ou doença reumatológica (Lúpus Eritematoso Sistêmico) - Estudo 1: Avaliação da resposta primária à vacinação

Pesquisadora responsável: Ana Marli Christovam Sartori

Instituição sede: Faculdade de Medicina da Universidade de São Paulo

<u>Introdução</u>: Pessoas vivendo com HIV/Aids, transplantados de órgãos sólidos e outros imunodeprimidos apresentam alto risco de infecção persistente pelo Papiloma Vírus Humano (HPV) e câncer relacionado ao HPV. Há três vacinas de HPV atualmente disponíveis: bivalente, quadrivalente e nonavalente. Poucos estudos avaliaram a imunogenicidade e a segurança da vacina de HPV em pessoas com comprometimento da imunidade.
<u>Objetivos</u>: O presente estudo avaliará a imunogenicidade e a segurança da vacina de HPV quadrivalente (HPV4V) em mulheres de 18 a 45 anos, em uso de imunossupressores devido a transplante de órgão sólido ou doença reumatológica em comparação a mulheres saudáveis da mesma faixa etária.
<u>Métodos</u>: Este ensaio clínico aberto incluirá 375 participantes, 125 mulheres transplantadas de órgão sólido (rim ou fígado), 125 mulheres com Lúpus Eritematoso Sistêmico e 125 mulheres imunocompetentes. Todas as participantes receberão três doses da vacina HPV4V (0, 2 e 6 meses) e serão acompanhadas por um período de sete meses (um mês após a terceira dose da vacina). A resposta primária à vacinação será avaliada um mês após a terceira dose (M7), pela frequência de soroconversão e título médio geométrico [GMT] de anticorpos antiHPV. A dosagem de anticorpos antiHPV será realizada por ensaio de anticorpos neutralizantes baseado em pseudovírion (*multiplexed pseudovirion-based serological assay* [PsV-Luminex]). Eventos adversos (EA) solicitados, locais e sistêmicos serão avaliados do momento da vacinação até sete dias após. EA não solicitados serão avaliados durante todo o período do estudo.

Descritores: vacinas contra papilomavírus, imunogenicidade, segurança, imunossupressão, transplante de órgão sólido, transplante renal, transplante hepático, doença reumatológica, lúpus eritematoso sistêmico

Abstract:

Project research title: Immunogenicity and safety of quadrivalent HPV vaccine in women using immunosuppressive drug due to solid organ transplant (kidney or liver) or rheumatologic disease (Systemic Lupus Erythematosus) –Study 1: Assessment of primary immune response to vaccination

Principal investigator: Ana Marli Christovam Sartori

Institution: Faculdade de Medicina da Universidade de São Paulo

Background. Persons living with HIV/aids, organ transplanted and other immunocompromised individuals have high risk of HPV persistent infection and HPV-related cancers. Three efficacious and safe HPV vaccines (bivalent, quadrivalent and nonavalent) are currently available. Few studies evaluated immunogenicity and safety of HPV vaccines in immunocompromised persons.
Objectives. The aim of this study is to evaluate immunogenicity and safety of quadrivalent HPV vaccine (HPV4V) in women aged 18 to 45 years, immunosuppressed due to solid organ transplant (kidney or liver) or Systemic Lupus Erythematosus, in comparison to healthy women of the same age.
Methods. This open label trial will include 375 participants, 125 kidney or liver transplanted women, 125 women with Systemic Lupus Erythematosus and 125 healthy (immunocompetent) women. All participants will receive three doses of HPV 4V vaccine (at 0, 2 and 6 months) and will be followed-up for seven months (one month after the third vaccine dose). Primary antibody responses to vaccination will be evaluated one month after the third vaccine dose (M7) by seroconversion rates and anti-HPV antibody geometric mean titers (GMT). A VLP specific multiplex HPV serological assay test (multiplexed pseudovirion-based serological assay [PsV-Luminex]) will be used to measure antiHPV antibodies. Solicited local and systemic adverse events (AE) following immunization will be accessed in all participants from the moment immediately after to 7 days after vaccine administration. Unsolicited AE will be evaluated during the entire study period.

Key words: HPV vaccine, immunogenicity, safety, immunosuppression, solid organ transplant, renal transplant, liver transplant, rheumatic disease, Systemic Lupus Erythematosus

1. Enunciado do problema:

1.1 Papiloma Vírus Humano

O Papilomavirus Humano (HPV) pertence ao gênero *Papillomavirus* da família *Papillomaviridae*. É um DNA vírus de dupla fita, não envelopado. A região não codificadora contribui para o controle da replicação do DNA e a produção das proteínas E1 a E7, L1 e L2. A proteína E5 está localizada na membrana celular e regula a transformação do receptor de fator de crescimento da epiderme, contribuindo para a oncogenicidade do HPV. As proteínas E6 e E7 dos HPVs oncogênicos apresentam capacidade de transformação através da ligação a receptores celulares e proteínas supressoras de tumores, além de inibirem a apoptose celular. O capsídeo viral é composto por duas proteínas, L1 e L2. Tipos distintos de HPV compartilham menos de 90% de semelhança na sequência de DNA que codifica L1.

Há mais de 100 tipos de HPV reconhecidos até o momento, sendo cada tipo relacionado a processos histopatológicos distintos. Os tipos de HPV são divididos em dois grupos: HPV de baixo risco, não oncogênicos, associados às verrugas genitais e papilomatose respiratória recorrente, doença rara, porém clinicamente significante; e os HPV de alto risco, oncogênicos, associados a cânceres cervical, vaginal, vulvar, peniano, anal e de orofaringe[1].

A infecção por HPV é a mais comum das doenças de transmissão sexual.

<u>HPV e câncer</u>

O HPV causa uma grande variedade de lesões e neoplasias, a maioria delas envolvendo a região anogenital. Infecção persistente por tipos oncogênicos de HPV é responsável pela quase totalidade dos cânceres de colo de útero. O câncer cervical representa cerca de 85% dos casos de câncer associado ao HPV[2]. O HPV é também responsável por 90% dos cânceres de ânus, 50% dos cânceres de pênis, 40% dos cânceres de vulva, 70% dos cânceres de vagina e 20-60% dos cânceres de orofaringe. Os HPV 16 e 18 são responsáveis por cerca de 70% dos cânceres cervicais e 80-90% dos cânceres de outros sítios anatômicos[3]. Os HPV não oncogênicos, principalmente os tipos 6 e 11, são associados a verrugas genitais e à papilomatose laríngea recorrente

A Organização Mundial de Saúde estima que, em 2012, ocorreram cerca de 445 mil novos casos e mais de 270 mil mortes por câncer cervical em todo mundo. Mais de 80% dos cânceres cervicais ocorrem em países não desenvolvidos, onde muitas mulheres não têm acesso aos programas de rastreamento[4]. Na América Latina, o câncer cervical é a principal causa de morte por neoplasia entre mulheres de 20 a 40 anos, e a terceira causa de câncer em mulheres, atrás apenas dos cânceres de mama e pulmão[5]. No Brasil, 18.503 novos casos e 8.414 mortes por câncer cervical foram notificados em 2012, sendo estimados 185.108 anos de vida perdidos[5].

HPV em imunodeprimidos

Pessoas vivendo com HIV/aids, assim como transplantados de órgãos e outros imunocomprometidos apresentam maior risco de infecção persistente e doença associada ao HPV[6]. Mulheres vivendo com HIV/aids têm risco de desenvolvimento de câncer cervical aumentado em 2-22 vezes em comparação a mulheres HIV negativas[6]. A prevalência de lesões precursoras de câncer cervical é também aumentada em transplantadas, mulheres com lúpus em uso de terapia imunossupressora, e outras doenças graves como insuficiência renal em diálise[7]. A imunodepressão é também associada ao desenvolvimento de câncer vulvar invasivo. Comparado à população geral, mulheres transplantadas têm risco de câncer vulvar aumentado em 100 vezes, risco esse que pode ser ainda maior que de mulheres vivendo com HIV/aids, na dependência da condição imunológica dessas últimas[6]. Câncer anal é um problema particular em homens que fazem sexo com homens soropositivos para HIV, que têm aumento de risco de cerca de 37 vezes[6]. Transplantados renais têm aumento de risco de desenvolvimento de câncer cervical de cerca de 14 vezes, de câncer vulvar de 50 vezes e de câncer anal de 100 vezes, quando comparados à população geral[8].

Em indivíduos imunodeprimidos, os cânceres tendem a ser mais agressivos que em pacientes imunocompetentes e são importante causa de morte nesta população. O risco aumentado de doença e morte por câncer após transplante de órgãos é atribuído à imunossupressão terapêutica crônica para evitar a rejeição do transplante[6]. Verrugas associadas ao HPV são também problema frequente em transplantados de órgãos[9; 10].

No Brasil, vários estudos avaliaram a prevalência da infecção por HPV na população geral e em pessoas vivendo com HIV/aids. Poucos estudos avaliaram a prevalência da infecção por HPV em mulheres com doenças reumatológicas em uso de tratamento imunossupressor. Um estudo em São Paulo encontrou menor frequência de infecção por HPV em mulheres com artrite reumatoide (14%), quando comparadas aos controles (30%)[11]. Também em São Paulo, um estudo encontrou maior prevalência de infecção por HPV em mulheres com lúpus (80,7%) em comparação ao grupo controle (35,7%)[12]. Outro estudo, no Rio de Janeiro, também encontrou maior prevalência de infecção por HPV em mulheres com lúpus (20,2%) em comparação aos controles (7,3%), sendo que o uso de terapia imunossupressora foi associado à infecção por HPV[13]. Na Bahia, um estudo encontrou maior frequência de condiloma vulvar em mulheres com lúpus em comparação aos controles (11.0 vs. 1.0 %, p <0.001)[14]. Não encontramos nenhum estudo avaliando a prevalência da infecção por HPV em transplantados de órgãos.

1.2 Vacinas de HPV

Composição

As vacinas de HPV são constituídas por proteínas estruturais do capsídeo viral (L1) recombinantes, que se juntam formando partículas virais vazias (*DNA-free virus-like particles,* VLP). Há três vacinas disponíveis comercialmente: uma vacina bivalente

(HPV 2V), que contém proteínas recombinantes do capsídeo (L1) dos HPV oncogênicos 16 e 18, responsáveis por cerca de 70% dos cânceres cervicais em todo o mundo; uma vacina quadrivalente (HPV 4V), que além dos HPV 16 e 18, contém também os tipos 6 e 11, não oncogênicos, responsáveis por 90% das verrugas genitais, e uma vacina nonovalente (HPV 9V), recentemente comercializada, que contém os HPV 6, 11, 16 e 18, e mais os tipos oncogênicos 31, 33, 45, 52 e 58 (Quadro 1)[3; 15]. Esses cinco tipos de HPV adicionais na vacina HPV 9V são responsáveis por cerca 18% dos cânceres cervicais, aumentando a cobertura da vacina para cerca de 90% dos cânceres cervicais. A vacina HPV 9V ainda não tem aprovação da ANVISA. As vacinas não contem timerosal, antibióticos ou outros preservativos[16].

Quadro 1. Vacinas de HPV atualmente disponíveis comercialmente

Vacina	Bivalente (HPV 2V)	Quadrivalente (HPV 4V)	Nonovalente (HPV 9V)
Nome, Produtor	Cervarix®, GSK	Gardasil®, Merck	Gardasil-9, Merck
Tipos HPV	HPV 16 e 18, oncogênicos	HPV 16, 18, oncogênicos<br>6 e 11, não oncogênicos	HPV 6, 11, 16, 18, 31, 33, 45, 52 e 58 oncogênicos
Dose proteína L1	20 / 20 µg	20 µg (HPV 6 e 18) e<br>40 µg (HPV 11 e 16)	30µg/40µg/60µg/40µg/ 20µg/20µg/20µg/20µg/20µg de HPV 6/11/16/18/31/33/ 45/52/58
Célula produtora	*Trichoplusia ni* (Hi 5), linhagem celular de inseto infectada com baculovírus com L1 recombinante	*Saccharomyces cerevisiae*	*Saccharomyces cerevisiae*
Adjuvante	500 µg AS04 (monofosforil lipid A + Hidroxido Alumínio)	225 µg Sulfato Alumínio	500 µg Sulfato Alumínio
Esquema vacinal	3 doses: 0, 1 e 6 meses, ou<br>2 doses: 0 e 6 meses, para adolescentes de 9 a 14 anos	3 doses: 0, 2 e 6 meses, ou<br>2 doses: 0 e 6 meses, para adolescentes de 9 a 14 anos	3 doses: 0, 2 e 6 meses

Imunogenicidade:

As vacinas de HPV induzem forte resposta de anticorpos IgG neutralizantes tipo-específicos. Em mulheres imunocompetentes, os títulos de anticorpos após

vacinação são até 100 vezes mais altos que após infecção natural. Há evidências de que os anticorpos séricos neutralizantes são responsáveis pela proteção induzida pela vacina[17]. IgG é o anticorpo predominante nas secreções vaginais e o mecanismo proposto de proteção é por transudação de IgG pelo epitélio vaginal[16]. Correlatos de proteção, isto é, a concentração mínima de anticorpos que esteja correlacionada com proteção clínica, não foram estabelecidos[3]. Porém, há evidências de que os títulos de anticorpos neuralizantes induzidos pela vacina são bem mais altos que os necessários para a proteção, uma vez que títulos mais baixos, induzidos por apenas uma dose da vacina bivalente, foram protetores em um estudo[2]

Eficácia:

As vacinas HPV 2V e HPV 4V foram testadas em mulheres de 15 a 26 anos, em ensaios clínicos de fase 3 que avaliaram, como desfecho, lesões precursoras de câncer cervical: neoplasia intraepitelial cervical (NIC) 2 e 3, adenocarcinoma in situ (AIS), NIC-1 e infecção persistente por HPV. Em seguimento de quatro anos, estas vacinas tiveram alta eficácia (90-100%) na proteção de novas infecções e lesões precursoras (NIC-2 e 3 e AIS) pelos tipos vacinais, em mulheres imunocompetentes sem infecção prévia por HPV (soronegativas e DNA negativas para os tipos de HPV vacinais, no momento da inclusão), que receberam pelo menos uma dose da vacina[2; 3; 18].

Proteção cruzada parcial contra lesões precursoras por outros tipos de HPV (não vacinais, 31 e 45) foi relatada, principalmente após a vacina HPV 2V[19].

A vacina HPV 4V teve também alta eficácia (>95%) na proteção de verrugas genitais pelos tipos vacinais[18].

A duração da proteção induzida pela vacina e a necessidade de doses de reforço ainda não são bem conhecidas. Em mulheres imunocompetentes, foi demonstrada persistência de anticorpos e eficácia por até oito anos após a vacinação[3; 18]. Resposta de anticorpos tipo "booster" à dose de reforço administrada cinco anos após a vacinação básica foi demonstrada para a vacina HPV 4V [3; 18].

Segurança

As duas vacinas apresentam excelente perfil de segurança. Reações no local de aplicação são relatados com as duas vacinas em frequência semelhante: dor, eritema e edema foram relatados, respectivamente, em até 80%, 25% e 25% dos vacinados, porém são de curta duração e resolvem espontaneamente. Eventos adversos sistêmicos também foram relatados, principalmente cefaleia e síncope imediatamente após vacinação, atribuída a síndrome vasovagal[16]. Como precaução, adolescentes vacinadas devem permanecer sentadas, em observação, por 15 minutos após a vacinação.

Até o final de 2013, mais de 144 milhões de doses da vacina HPV 4V e 41 milhões de doses da vacina HPV 2V haviam sido distribuídas no mundo todo. Os eventos adversos mais relatados foram reações no local de aplicação, cefaleia e tonturas. Síncope após a vacinação é um dos eventos adversos mais relatados[3]. Na literatura, há evidências da segurança e imunogenicidade da vacina HPV4v em meninas e mulheres imunocompetentes de 9 a 45 anos[20]. No Brasil, a vacina HPV4v é aprovada pela

Agência Nacional de Vigilância Sanitária (ANVISA) para uso em mulheres de 9 a 45 anos e em homens de 9 a 26 anos[21].

Não há evidências consistentes de associação da vacina com doenças autoimunes, como síndrome de Guillain-Barré, doenças desmielinizantes, tireoidite autoimune ou doenças to tecido conectivo (lúpus, artrite reumatóide, ou artrite juvenil)[3].

Uso da vacina de HPV em imunodeprimidos

Vários estudos avaliaram a imunogenicidade e segurança das vacinas de HPV 2V e HPV 4V em pessoas vivendo com HIV/aids[22]. Uma revisão recente dos estudos publicados concluiu que ambas as vacinas parecem seguras e imunogênicas, com altas taxas de soroconversão nesta população (crianças, adolescentes e adultos, de ambos os sexos, HIV+). Entretanto, os títulos de anticorpos são, em geral, mais baixos que os de pessoas HIV negativas (particularmente para o HPV 18)[22]. Não foram publicados estudos de seguimento a longo prazo das coortes HIV+ vacinadas e, portanto, a duração da resposta de anticorpos não é conhecida nesta população[22].

Poucos estudos avaliaram a imunogenicidade e segurança das vacinas de HPV em outros imunodeprimidos[23; 24; 25; 26; 27; 28; 29]. Um estudo avaliou a vacina HPV 4V, em esquema de três doses (0, 2 e 6 meses) em um pequeno grupo de transplantados renais (n=14) e hepáticos (n=3), de ambos os sexos, de 9 a 17 anos. Todos os transplantados renais e dois transplantados hepáticos eram negativos para os quatro tipos de HPV contidos na vacina. Nove participantes (7 transplantados renais e 2 transplantados hepáticos) completaram o esquema vacinal. Um mês após a terceira dose da vacina, todos os 9 apresentaram soroconversão para os quatro tipos de HPV vacinais. Eventos adversos foram leves e incluíram dor e edema no local da vacinação, febre, cefaleia, tosse, pneumonia e diarreia[23].

Um estudo canadense avaliou a vacina HPV 4V em 47 homens e mulheres transplantados de órgãos sólidos (rim, pulmão, fígado ou coração), de 18 a 35 anos, que receberam esquema de três doses (0, 2 e 6 meses). A imunogenicidade foi avaliada por teste de ELISA, realizado quatro semanas após a terceira dose. Anticorpos específicos para os HPV 6, 11, 16 e 18 foram detectados em, respectivamente, 63,2%, 64,8%, 63,2% e 52,6% dos participantes. Houve significativa redução dos títulos de anticorpos aos 12 meses após a vacinação. A vacina foi bem tolerada; dor no local da vacinação, febre subjetiva, tonturas e cefaleia foram observadas após a primeira dose, com poucos eventos adversos relatados nas doses subsequentes[24].

Uma revisão sistemática recente encontrou cinco estudos em pacientes com doenças autoimunes: três estudos avaliaram as vacinas de HPV em pacientes com lúpus (n=83); dois estudos avaliaram pacientes com artrite juvenil (n=74); e um estudo avaliou pacientes com doença inflamatória intestinal (n=37), totalizando 194 sujeitos[25]. Em dois estudos, que avaliaram a vacina HPV 4V em pacientes com lúpus, a frequência de soroconversão após a vacinação foi semelhante à dos controles saudáveis[27; 28]. Entretanto, os títulos de anticorpos foram mais baixos nos participantes com lúpus em uso de terapia imunossupressora[27; 29]. Os dois estudos que avaliaram pessoas com artrite juvenil relataram soroconversão após a terceira dose da vacina em todos os sujeitos, porém com títulos de anticorpos mais baixos que nos controles saudáveis[28; 29]. Um

estudo incluiu 37 indivíduos de 9 a 26 anos com doença inflamatória intestinal (doença de Crohn, retocolite ulcerativa ou colite indeterminada) imunossuprimidas. Após três doses da vacina HPV 4V, todos soroconverteram para os HPV 6, 11 e 16; dois pacientes não soroconverteram para HPV 18[26]. As vacinas de HPV foram bem tolerada em todos estes estudos. Aumento da atividade da doença de base após a vacinação não foi observado em dois estudos, que incluíram pacientes com lúpus e com artrite juvenil. No estudo que avaliou pacientes com doença inflamatória intestinal, ocorreram duas hospitalizações por exacerbação da doença de base; entretanto o estudo não tinha controles não vacinados, o que impede qualquer conclusão[25].

A resposta dos imunodeprimidos à vacinação pode ser diferente de acordo com o grau de imunocomprometimento. Pessoas vivendo com HIV/Aids em uso de terapia antirretroviral de alta eficácia apresentam resposta imune às vacinas semelhante a pessoas imunocompetentes soronegativas[30; 31; 32]. Por outro lado, pessoas submetidas a transplante de órgãos sólidos ou portadoras de doença autoimune podem necessitar de uso prolongado de imunossupressores, muitas vezes por toda a vida, tendo maior impacto na resposta à vacina.

Uso da vacina no Brasil:

A vacina HPV 4V foi introduzida no calendário de vacinação de adolescentes do Programa Nacional de Imunizações (PNI), em esquema de duas doses (0 e 6 meses). No primeiro ano do programa (2014), foram vacinadas meninas de 11 a 13 anos, no segundo ano (2015), meninas de 9 a 13 anos, e, a partir de 2016, serão vacinadas meninas de 9 anos. A vacinação é feita em escolas, em duas campanhas anuais (março e setembro). A vacina é também disponível nas unidades básicas de saúde (UBS) durante todo o ano[33]. A cobertura vacinal da segunda dose da vacina em meninas de 11 a 13 anos foi de 85%, nos dois primeiros anos do programa de vacinação[34].

Desde março de 2015, a vacina de HPV 4V é disponível, em todas as salas de vacina, também para mulheres vivendo com HIV/aids de 14 a 26 anos, em esquema de três doses (0, 2 e 6 meses)[35].

Em 2017, o PNI passou a recomendar a vacina HPV4v também para meninos de 12 a 13 anos de idade. A faixa etária será ampliada, gradativamente, até 2020, quando serão incluídos os meninos com 9 anos até 13 anos de idade. Os homens de 14 a 26 anos vivendo com HV/aids também receberão a vacina. A vacina HPV4v também estará disponível nos Centros de Referência para Imunobiológicos Especiais (CRIEs) para imunodeprimidos (indivíduos submetidos a transplantes de órgãos sólidos, transplantes de medula óssea ou pacientes oncológicos), de ambos os sexos, de 9 a 26 anos de idade, que deverão receber esquema de três doses (0, 2 e 6 meses)[36].

2. Justificativa para realização do estudo:

Embora já em uso rotineiro no Programa Nacional de Imunização no Brasil e em muitos outros países, há vários aspectos da vacina de HPV que merecem novos estudos, em particular em relação ao seu uso em imunodeprimidos. Poucos estudos avaliaram a

segurança e imunogenicidade da vacina nesta população. Os estudos de persistência de anticorpos e duração da proteção clínica a mais longo prazo (8 anos) são restritos às mulheres imunocompetentes, havendo poucos estudos de persistência de anticorpos em imunodeprimidas com avaliação até 12 meses após a vacinação. Não há estudos da eficácia da vacina em imunodeprimidos. No Brasil, poucos estudos avaliaram a soroprevalência e a prevalência de infecção por HPV em mulheres com doença reumatológica. Não encontramos nenhum estudo conduzido no Brasil sobre a soroprevalência / prevalência de infecção por HPV em mulheres transplantadas de órgãos sólidos.

3. Objetivos:

1. Avaliar a resposta imune primária e a segurança da vacina de HPV 4V em mulheres de 18 a 45 anos imunossuprimidas transplantadas de órgãos sólidos (rim ou fígado) ou com doença reumatológica (lúpus eritematoso sistêmico), sem história prévia de doença associada ao HPV (câncer ou lesões precursoras – NIC-2, NIC-3, AIS);

2. Comparar a resposta imune primária e a segurança da vacina de HPV 4V em mulheres de 18 a 45 anos transplantadas (de rim e fígado) e em mulheres imunocompetentes da mesma faixa etária;

3. Comparar a resposta imune primária e a segurança da vacina de HPV 4V em mulheres de 18 a 45 anos com lúpus eritematoso sistêmico e em mulheres imunocompetentes da mesma faixa etária;

4. Avaliar a soroprevalência e prevalência de HPV, assim como a frequência dos diferentes tipos de HPV e de lesões cervicais associadas ao HPV, em mulheres de 18 a 45 anos em uso de terapia imunossupressora devido transplante de órgãos sólidos (rim ou fígado) ou com doença reumatológica (lúpus eritematoso sistêmico) (no momento de inclusão ao estudo).

5. Avaliar fatores de risco para a infecção por HPV nesta população de mulheres de 18 a 45 anos em uso de terapia imunossupressora devido transplante de órgãos sólidos (rim ou fígado) ou com doença reumatológica (lúpus eritematoso sistêmico)

4. Métodos:

4.1 Desenho do estudo:

Este será um ensaio clínico aberto, que avaliará a segurança e a imunogenicidade da vacina de HPV quadrivalente (HPV 4V) em mulheres de 18 a 45 anos em uso de terapia imunossupressora, transplantadas de órgãos sólidos (rim ou fígado) ou com doenças reumatológicas (lúpus eritematoso sistêmico), em comparação a mulheres saudáveis da mesma faixa etária.

Cada um desses grupos será comparado ao grupo controle de forma independente. Serão incluídos 375 participantes, sendo 125 transplantadas de órgãos sólidos (rim ou

fígado); 125 com doença reumatológica (lúpus eritematoso sistêmico) e 125 mulheres saudáveis. Todas as participantes receberão três doses de vacina de HPV 4V (esquema vacinal – 0, 2 meses e 6 meses) e serão acompanhadas por um período de sete meses (um mês após a terceira dose da vacina). Serão avaliados desfechos de segurança e imunogenicidade nos três grupos.

4.2 População do estudo:

Mulheres de 18 a 45 anos poderão participar do estudo. Para serem incluídas, devem preencher todos os critérios de inclusão e não se enquadrarem em nenhum dos critérios de exclusão.

4.2.1 Critérios de inclusão:

As potenciais participantes somente serão incluídas no estudo se preencherem todos os critérios abaixo:

- Sexo feminino
- Idade de 18 a 45 anos
- Estar disponível para participar durante todo o período do estudo
- Demonstrar interesse de participar do estudo, documentada pela assinatura do TCLE (ANEXO 1)

No grupo de transplantadas:

- Ter decorrido pelo menos seis meses do transplante de órgão (rim ou fígado)
- Estar em uso de droga imunossupressora

No grupo de mulheres com lúpus eritematoso sistêmico:

- Estar em uso de drogas imunossupressoras
- Estar em uso de esquema terapêutico contendo cloroquina

No grupo de mulheres imunocompetentes:

- Mulheres sem doenças imunodepressoras e que não façam uso de corticosteroides ou quaisquer outras drogas imunossupressoras

4.2.2 Critérios de exclusão

As participantes em potencial que preencherem qualquer um dos critérios abaixo serão excluídos no estudo:

- Gestação
- Amamentação;
- Outras doenças com comprometimento imunológico: HIV/Aids: *diabetes mellitus*, neoplasias ou imunodeficiências congênitas;

- Quimioterapia ou radioterapia (para tratamento de câncer) nos seis meses anteriores à inclusão ao estudo;
- Uso de terapia contendo imunobiológico;
- Doença ativa grave com comprometimento neurológico, cardíaco, pulmonar, hepático ou renal;
- Doença comportamental, cognitiva ou psiquiátrica que, na opinião dos investigadores afete a capacidade de entender e colaborar com as exigências do protocolo de pesquisa;
- Dependência de álcool ou drogas;
- Doença aguda ou febre no momento da inclusão;
- História de vacinação de HPV;
- Ter recebido qualquer outra vacina até 30 dias antes da inclusão no estudo;
- Ter recebido qualquer hemoderivado nos últimos seis meses;
- História de qualquer lesão cervical, vulvar ou vaginal relacionada ao HPV;
- História de verruga genital;
- História de alergia a qualquer componente da vacina;
- Qualquer outra condição que a julgamento do investigador poderia vir a prejudicar os procedimentos de estudo como: risco de perda do órgão transplantado.

4.2.3 População fonte do estudo e área de recrutamento

As participantes serão recrutadas nos ambulatórios das Divisões de Ginecologia e Reumatologia e dos Transplantes renal e hepático, do HC-FMUSP. As mulheres imunocompetentes serão recrutadas no Centro de Referência para Imunobiológicos Especiais do HCFMUSP.

4.3 Desfechos do estudo

4.3.1. Desfecho primário de imunogenicidade:

Será avaliado para cada tipo de HPV incluído na vacina.

- Resposta humoral primária à vacinação será avaliada 1 mês após a terceira dose da vacina (M7) por:
  - Frequência de soroconversão
  - Título médio geométrico (GMT) de anticorpos

A dosagem de anticorpos antiHPV será realizada por ensaio de anticorpos neutralizantes baseado em pseudovírion (*multiplexed pseudovirion-based serological assay, PsV-Luminex*), que detecta anticorpos neutralizantes contra 15 tipos de HPV, entre os quais os tipos de HPV contidos na vacina 4V (6, 11, 16 e 18), os tipos incluídos apenas na vacina HPV 9V (31, 33, 45, 52 e 58) e outros tipos não contidos em nenhuma das vacinas atuais (35, 39, 56, 59, 68, 73) [37; 38]. A realização de teste sorológico para os tipos de HPV contidos na vacina 9V permitirá avaliar potencial benefício desta vacina para esta população.

O teste sorológico será realizado no Department of Laboratory Medicine, Karolinska Institute, Suécia.

4.3.2. Desfecho primário de segurança:

Serão avaliados os eventos adversos locais e sistêmicos, solicitados e não solicitados, após a vacinação de todos os participantes do momento imediatamente após a vacinação até 7 dias depois:

- Todos os participantes deverão permanecer no serviço por 30 minutos após cada dose de vacina, para avaliação de eventos adversos imediatos
- Será solicitado que todos os participantes anotem no diário, detalhadamente, sinais e sintomas que ocorrerem no período de 7 dias após a vacinação. Será solicitado a todos os participantes que tragam o diário preenchido na visita seguinte para que qualquer informação adicional seja adequadamente adicionada ao e-CRF.
- Será fornecido um termômetro para cada participante e será solicitado que anote a temperatura 1 vez ao dia ou se tiver febre, anotando o resultado no diário do participante
- Todos os participantes serão contatados por telefone, correio eletrônico ou mensagem de texto (SMS, WhatsApp) três dias após cada dose de vacina e será aplicado questionário estruturado. O objetivo deste contato é de reduzir a perda de informação devido a perda do diário de sintomas e monitorar qualquer sinal relacionado à segurança da vacina.
- Informações sobre eventos adversos graves serão monitorados durante todo o estudo.
- Rejeição ao órgão transplantado será monitorado durante todo o estudo, conforme rotina de cada serviço (aumento de creatinina nos transplantados renais e aumento de enzimas hepáticas nos transplantados de fígado e biópsia, se necessária).
- A atividade lúpica será monitorada durante todo o estudo pelo SLEDAI (*Systemic Lupus Erythematosus Disease Activity Index)*.

Neste estudo, eventos adversos solicitados serão:

- Locais: dor, edema e eritema
- Sistêmicos: síncope, febre, cefaleia, gastroenterite (diarreia)

Os eventos adversos solicitados e não solicitados serão avaliados pelos investigadores em relação a:

- Gravidade e intensidade
- Relação causal com a vacina
- Medidas tomadas
- Evolução do quadro

A classificação da intensidade dos eventos adversos solicitados e não solicitados será feita de acordo com o Quadro 2. O Quadro 3 mostra a classificação dos eventos adversos de acordo com a associação com a vacinação.

Quadro 2. Classificação dos eventos adversos locais e sistêmicos, conforme intensidade (adaptado de FDA, 2007)[39].

**Eventos adversos locais**				
	Leve (grau 1)	Moderado (grau 2)	Grave (grau 3)	Com potencial de ameaça a vida (grau 4)
Dor	Não interfere na atividade diária	Uso repedido de analgésico não narcótico por >24 horas ou interfere pouco na atividade diária	Uso de analgésico narcótico ou impede a atividade diária	Visita ao pronto socorro ou hospitalização
Sensibilidade	Leve desconforto ao toque	Desconforto com movimentação	Desconforto significativo em repouso	Visita ao pronto socorro ou hospitalização
Eritema	2,5 a 5,0cm	5,1 a 10m	>10cm	Necrose ou dermatite esfoliativa
Enduração/edema	2,5 a 5,0cm e não interfere nas atividades diárias	5,1 a 10m e interfere nas atividades diárias	>10cm ou impede atividades diárias	Necrose
**Eventos adversos sistêmicos**				
Febre	38,0 a 38,4°C	38,5 a 38,9ºC	39,0 a 40,0ºC	>40ºC
Náuseas/vômitos	Não interfere nas atividades ou ≤2 episódios/24 horas	Alguma interferência nas atividades ou >2 episódios/24 horas	Impede atividades diárias e necessita de hidratação endovenosa	Visita ao pronto socorro ou hospitalização por hipotensão
Diarreia	2 a 3 evacuações ou <400g/24 horas	4 a 5 evacuações ou 400g a 800/24 horas	≥6 evacuações ou >800g/24 horas ou necessita de hidratação endovenosa	Visita ao pronto socorro ou hospitalização por hipotensão
Cefaleia	Não interfere com as atividades diárias	Uso repedido de analgésico não narcótico por >24 horas ou alguma interferência na atividade diária	Uso de analgésico narcótico ou impede a atividade diária	Visita ao pronto socorro ou hospitalização
Outros eventos adversos	Não interfere com as atividades diárias; causa desconforto leve, é bem tolerado e desaparece espontaneamente	Alguma interferência na atividade diária	Impede a atividade diária	Potencialmente fatal. Visita ao pronto socorro ou hospitalização

Quadro 3. Classificação dos eventos adversos de acordo com a associação com a vacinação[40].

	Consistente	Indeterminada	Inconsistente/Coincidente
**Informação adequada disponível**	A1: reações inerentes ao produto, conforme literatura	B1: relação temporal, mas sem evidências na literatura para de estabelecer relação causal	C1: condições preexistentes ou emergentes
	A2: reações inerentes à qualidade do produto	B2: os dados da investigação são conflitantes em relação à causalidade	C2: condições causadas por outros fatores e não por vacinas
	A3: erros de imunização		
	A4: reações de ansiedade relacionadas à imunização		
**Informação adequada indisponível**	D: inclassificável<br>Especificar informações adicionais relacionadas à imunização		

4.3.3. Desfechos secundários:

Soroprevalência de HPV (infecção prévia)

Será avaliada no momento da inclusão ao estudo, através do ensaio de anticorpos neutralizantes baseado em pseudovírion (*PsV-Luminex*).

Prevalência de infecção por HPV atual (no momento da inclusão)

Será avaliada por detecção / genotipagem de HPV por técnica de PCR. As amostras cervicais serão testadas para presença de HPV e genotipagem pelo método PapilloCheck®, baseado em Reação de Polimerase em Cadeia. Este teste detecta 24 genótipos de HPV, incluindo os sorotipos incluídos na vacina HPV4V (6, 11, 16, 18), os sorotipos incluídos apenas na vacina HPV9V (31, 33, 45, 52 e 58) e os seguintes sorotipos não vacinais 35, 39, 40, 42, 43, 44, 51, 53.56, 59, 66, 68, 70, 73 e 82 [41]. O exame será realizado no momento de inclusão (M0), no Laboratório de Virologia do Instituto de Medicina Tropical (LIM-52).

Prevalência de lesões cervicais associadas ao HPV

Será avaliada por esfregaço cervicovaginal pelo método de citologia em base líquida, que será realizada no momento de inclusão ao estudo (M0) de acordo com as diretrizes do Ministério da Saúde para rastreamento de câncer de colo de útero, segundo as quais o Papanicolaou deve ser realizado a partir do início da atividade sexual [42].

Mulheres que não iniciaram a vida sexual poderão ser incluídas no estudo, mas não farão exame ginecológico ou coleta de material cervical para citologia e detecção/ genotipagem de HPV.

Nos casos em que for detectada alteração do exame colpocitológico, será feito manejo clínico de acordo com as recomendações do Ministério da Saúde[42].

4.4. Variáveis de interesse

Será feita entrevista com as participantes no momento de inclusão e consulta aos prontuários médicos para coleta de dados demográficos (idade, etnia, escolaridade, renda, estado civil), hábitos (tabagismo; etilismo); práticas sexuais (idade de início da vida sexual; número de parceiros em toda vida, nos últimos cinco anos e no último ano; frequência de relações sexuais; prática de sexo oral e anal), saúde reprodutiva (uso de anticoncepcional oral, dispositivo intrauterino [DIU] e condom; número de gestações, partos e abortos); fase do ciclo sexual no momento do estudo e dados clínicos referentes à doença de base e/ou transplante, comorbidades e drogas imunossupressoras e outros medicamentos em uso.

4.5. Procedimentos para realização do estudo

Os participantes em potencial serão atendidos inicialmente no CRIE HCFMUSP ou no ambulatório de Ginecologia do HCFMUSP pela equipe de pesquisadores.

Inclusão: procedimentos que poderão ser realizados em período de até 14 dias antes do D0:

- Fornecer informações sobre os objetivos e procedimentos do estudo e aplicar o Termo de Consentimento Livre e Esclarecido (TCLE)
- Verificar os critérios de inclusão e exclusão
- Realizar anamnese e exame clínico
- Coletar material para citologia em base líquida
- Coletar material para detecção/genotipagem de HPV
- Coletar sangue para realização de sorologia de HPV
- Realização de teste rápido de HIV

Dia 0: procedimentos a serem realizados

- Realização de teste de gravidez
- Vacinação: 1ª dose
- Observação por 30 minutos após vacinação
- Entrega do diário para anotação de eventos adversos

Mês 2 (+- 7 dias): procedimentos a serem realizados

- Entrevista sobre eventos adversos após a 1ª dose com recolhimento do diário
- Realização de teste de gravidez
- Vacinação: 2ª dose
- Observação por 30 minutos após vacinação
- Entrega do diário para anotação de eventos adversos

Mês 6 (+- 14 dias): procedimentos a serem realizados

- Entrevista sobre eventos adversos após a 2ª dose com recolhimento do diário
- Realização de teste de gravidez
- Vacinação: 3ª dose
- Observação por 30 minutos após vacinação
- Entrega do diário para anotação de eventos adversos

Mês 7 (+- 7 dias): procedimentos a serem realizados

- Entrevista sobre eventos adversos após a 3ª dose com recolhimento do diário
- Coleta de amostra de sangue para realização da sorologia
- Realizar anamnese e exame clínico

4.6. Critérios de retirada do estudo

Os participantes serão retirados do estudo, pelos investigadores, se ocorrer qualquer uma das circunstâncias abaixo:

- Retirada do consentimento pelo participante
- Contraindicação da segunda ou terceira dose da vacina de HPV
- Evento adverso grave
- Descumprimento do protocolo
- Óbito
- Erro de seleção: da perspectiva de análise, "erro de seleção" é qualquer participante que foi incluído no estudo, porém retirado antes da vacinação por violação do protocolo (critérios de inclusão/exclusão)
- Perda de seguimento: qualquer participante com pelo menos uma visita (exemplo: pelo menos uma dose de vacina), porém com perda do seguimento (sem informação de segurança ou sem desfecho de eficácia disponível)

4.7. Metodologia laboratorial:

4.7.1. Detecção e dosagem de anticorpos contra HPV:

A dosagem sérica de anticorpos anti-HPV será realizada por ensaio de anticorpos neutralizantes baseado em pseudovírion (*multiplexed pseudovirion-based serological assay, PsV-Luminex*)[37; 38; 43; 44]. Os soros serão analisados na diluição 1:150. Valores cut-off para definir soropositividade serão calculados independentemente para

cada tipo de HPV, pela análise da média dos valores de unidade de intensidade de fluorescência (MFI) obtidos de soros de crianças brasileiras, de 2 a 10 anos de idade. As amostras de soro de crianças brasileiras serão obtidas do banco de amostras do protocolo de pesquisa "Estudo da incidência de Dengue no Brasil em municípios de alta e média endemicidade: Goiânia-GO e Araraquara-SP" (Laboratório de Virologia - LIM-52). Será utilizado algoritmo para o cut-off como recomendado pelo Global HPV LabNet: valor médio MFI de um painel de soros controles negativos mais 3 desvios-padrão[43]. Se este valor de cut-off for excessivamente baixo (<400 MFI), será utilizado 400 MFI como cut-off para ter sensibilidade e especificidade semelhantes ao ELISA clássico[38]. Os títulos de anticorpos específicos antiHPV 16 serão convertidos em unidades internacionais (UI) usando Soro Padrão Internacional para HPV 16 (10UI) e wPLL (*a least squares-weighted modification of the parallel line model*)[44].

A dosagem de anticorpos será realizada antes da vacinação (D0), e um mês (+/-7 dias) após a terceira dose (M7).

A sorologia de HPV será realizada no Departament of Laboratory Medicine, Karolinska Institute, Suécia.

4.7.2. Detecção e genotipagem de HPV

As amostras cervicais serão coletadas em meio de citologia líquida e guardadas (de 2 a 8°C) por no máximo três semanas, e encaminhadas para o laboratório. As amostras serão centrifugadas e a extração de DNA realizada a partir do pellet de células ressuspensas no próprio meio de transporte utilizando QIAamp DNA Mini Kit (Qiagen, Gaithersburg, USA), de acordo com as instruções do fabricante. Para cada reação, 5 µl da solução do DNA será utilizado para detecção e genotipagem de HPV com o PapilloCheck®, que é baseado na amplificação por PCR de um fragmento de aproximadamente 350bp da região E1 do genoma do HPV[41]. A pesquisa de HPV será realizada antes da vacinação (D0).

A detecção e genotipagem de HPV serão realizadas no Laboratório de Virologia do Instituto de Medicina Tropical da USP (IMT-USP).

4.7.3. Armazenamento do material biológico

As amostras biológicas serão armazenadas no Laboratório de Virologia do Instituto de Medicina Tropical (IMT-USP). As amostras cervicais serão armazenadas a 4°C até a extração de DNA. O DNA extraído ficará a -20°C. O plasma para sorologia será estocado a -20°C. Após o término do estudo, as amostras serão encaminhadas para o Biobanco do IMT-USP.

Quadro 4: Procedimentos do estudo

	Triagem e inclusão (até 14 dias antes D0)	Dia 0	Mês 2 ±7 dias	Mês 6 ±14 dias	Mês7 ±7 dias
**Aplicação e assinatura do TCLE**	X				
**Critérios inclusão/exclusão**	X				
**História Médica**	X		X	X	X
**Exame físico**	X		X	X	X
**Teste rápido de HIV**	X				
**Coletar material para colpocitológico**	X				
**Coletar amostra cervical para pesquisa de HPV**	X				
**Coletar sangue para sorologia de HPV**	X				X
**Coleta de sangue (volume)**	10 ml		10 ml		10 ml
**Teste de gravidez**		X	X	X	
**Vacinação – HPV 4V**		X	X	X	
**Entrega de diário de eventos adversos**		X	X	X	

4.8. Tamanho da amostra e poder estatístico

4.8.1. Cálculo da amostra

Foram calculados os tamanhos das amostras para comparação da proporção de soroconversão de cada um dos grupos de imunodeprimidas, para cada tipo de HPV contido na vacina, com o grupo controle. O cálculo da amostra considerou comparação de duas proporções, com proporção de 1:1 entre o grupo controle e cada um dos grupos de mulheres imunodeprimidas, poder do estudo de 80% e 90%, e erro tipo 1 (α) de 5%. Foram consideradas como referência de soroconversão, em mulheres com lúpus eritematoso sistêmico, os valores encontrados por Mok e cols[27] e, em mulheres transplantadas, por Kumar e cols[24]. Os cálculos foram feitos no site http://powerandsamplesize.com/Calculators/Compare-2-Proportions/2-Sample-Equality, que utiliza a seguinte fórmula para o cálculo de tamanho amostral:

$$n_A = \kappa n_B \ \text{and} \ n_B = \left(\frac{p_A(1-p_A)}{\kappa} + p_B(1-p_B)\right)\left(\frac{z_{1-\alpha/2} + z_{1-\beta}}{p_A - p_B}\right)^2$$

Onde:
$\kappa = n_A / n_B$ a proporção entre os dois grupos
Φ é a função de distribuição normal padrão
Φ^{-1} é a função do quantil normal padrão
α é o erro tipo I
ß é o erro tipo II, sendo o poder 1- ß

Os Quadros 5 e 6 mostram o tamanho das amostras encontradas para cada um dos grupos, conforme soroconversão observada para os diferentes tipos de HPV.

Quadro 5: Tamanho amostral calculado para cada tipo de HPV em mulheres com doença autoimune em uso de imunossupressor.

		HPV 6 (%)	HPV 11 (%)	HPV 16 (%)	HPV 18 (%)
Proporção de soroconversão após vacinação[27]	Lúpus	82	89	95	76
	Controles	98	98	98	80
Nº de participantes em cada grupo	(β= 80%)	52	114	584	1677
	(β= 90%)	69	153	782	2245

Quadro 6: Tamanho amostral calculado para cada tipo de HPV em mulheres transplantadas.

		HPV 6 (%)	HPV 11 (%)	HPV 16 (%)	HPV 18 (%)	
Proporção de soroconversão após vacinação	Transplantadas[24]	63	68	63	52	88*
	Saudáveis	95	95	95	80	98
Nº de participantes em cada grupo	(β= 80%)	22	29	22	41	99
	(β= 90%)	29	39	29	55	132

* Valor assumido considerando -10% como diferença clinicamente significativa e 98% de soroconversão como referência para mulheres saudáveis.

Considerando como clinicamente não significativas as diferenças de soroconversão menores de 5% (HPV 16 e 18), consideramos os dados referente à soroconversão para o HPV tipo 11 nas pacientes com lúpus como referência para este grupo. Considerando o cálculo para β= 80%, e assumindo 10% de perdas, foi adotado tamanho de amostra de 125 (114 + 11) mulheres em cada um dos grupos (com doença

reumatológica e saudáveis) como número capaz de detectar diferença de soroconversão clinicamente significativa entre os grupos.

Em relação às transplantadas de órgãos sólidos, as taxas de soroconversão observadas no estudo de Kumar e cols foram bem mais baixas do que nas mulheres com lúpus[24]. Entretanto, este estudo não tem controle interno, e usamos taxas teóricas de soroconversão em mulheres saudáveis para as estimativas. Além disso, em outro estudo que avaliou pequeno número de mulheres transplantadas[23], a taxa de soroconversão foi de 100%. Por essas razões, calculamos também a amostra capaz de detectar uma diferença de 10% na taxa de soroconversão, tendo como referência para o grupo saudável, 98% de soroconversão. Finalmente, para facilitar os procedimentos do estudo, optamos por adotar o mesmo tamanho amostral do grupo de pacientes com doença reumatológica que nos permite detectar diferenças nas taxas de soroconversão um pouco menores, porém clinicamente significativas.

4.9. Análise estatística

Os dados coletados serão inseridos em banco de dados eletrônico que será construído no REDCap (Research Eletronic Data Capture) [https://redcap.vanderbilt.edu/].

4.9.1. Análise de imunogenicidade

A resposta primária à vacinação será avaliada pela proporção de participantes que apresentarem soroconversão para cada tipo vacinal de HPV (6, 11, 16 e 18), quatro semanas ($\pm$ 7 dias) após a terceira dose de vacina; e pelo título médio geométrico (GMT) de anticorpos contra os quatro tipos vacinais de HPV (6, 11, 16 e 18), quatro semanas ($\pm$ 7 dias) após a terceira dose de vacina. Cada tipo de HPV será analisado separadamente. Participantes com PCR positivo ou soropositivas no momento da inclusão serão excluídas da análise para o tipo específico, porém serão incluídas na análise dos demais tipos virais para as quais eram negativas. Este procedimento já foi adotado em outros estudos de imunogenicidade da vacina de HPV[25,26]. Será feita análise descritiva da resposta de anticorpos para o tipo específico das participantes soropositivas no momento da inclusão.

A proporção de soroconversão e o respectivo Intervalo de Confiança (IC) de 95% serão comparados usando o teste Chi-quadrado (χ^2) ou o teste exato de Fisher, conforme apropriado. O GMT será calculado com IC de 95% e será comparado usando o teste *t*-Student.

A análise estatística será feita para cada um dos grupos – lúpus e transplantadas – separadamente. Para o grupo de transplantadas de órgãos sólidos, a análise será feita para o grupo como um todo, procedimento adotado anteriormente por outros autores[22]. Será feita análise descritiva da resposta imune à vacina de cada um dos grupos de transplantadas (rim e fígado) separadamente.

Para identificar variáveis de interesse possivelmente associadas à soroconversão, incluindo características demográficas, o tipo de drogas imunossupressoras em uso e o tipo de transplante (renal ou hepático), será utilizada regressão logística.

População de eficácia por protocolo (PP)

Esta análise consistirá de todas as participantes negativas no PCR e soronegativas para o tipo específico de HPV no momento de inclusão, que receberem as três doses de vacina, nos tempos definidos pelo protocolo, e tiverem a análise do título de anticorpos em suas amostras de sangue realizadas em quatro semanas (+7 dias) após a terceira dose de vacina de HPV. Participantes com desvio de protocolo graves ou interrupção do tratamento precoce por motivo não relacionado ao medicamento do estudo serão excluídas da análise PP. Desvio de protocolo grave é definido como falha ao preencher os critérios de inclusão e exclusão, não cumprimento do esquema de doses ou visitas conforme o protocolo.

População da análise por intenção de tratamento (IT)

Esta análise será o mais completa possível. A população da IT incluirá todas as participantes que receberem pelo menos uma dose da vacina do estudo e para as quais se tenha dados de acompanhamento das variáveis primárias, independente do cumprimento do calendário do protocolo.

4.9.2. Análise de segurança:

Eventos adversos locais e sistêmicos solicitados: serão listados, individualmente para cada participante, no dia da vacinação (dia 1) e nos sete dias subsequentes (dia 2 a dia 7) e resumidos de acordo com a frequência e intensidade, por grupo de tratamento, em tabelas e gráficos. A comparação de sujeitos que apresentarem evento adverso solicitado após cada dose de vacina em cada grupo de tratamento será realizado pelo teste chi-quadrado ou pelo teste exato de Fisher, de acordo como o mais apropriado.

Eventos adversos não solicitados serão listados individualmente para cada participante. Serão apresentados resumos com números e tipos de eventos e número de participantes que experimentaram eventos adversos não solicitados durante todo o estudo. A comparação dos eventos adversos não solicitados entre os grupos será apenas descritiva.

A população de segurança incluirá todos os participantes que receberam pelo menos uma dose da vacina para os quais houver informações.

5. Questões éticas

Este projeto será encaminhado para aprovação pelo Comitê de Ética em Pesquisa do Hospital das Clínicas da Faculdade de Medicina da Universidade de São Paulo (CAPPesq). O projeto só será iniciado após aprovação pelo Sistema CEP.

As participantes serão incluídas após assinatura do Termo de Consentimento Livre e Esclarecido (TCLE, Anexos 1 e 2).

A coleta e processamento de dados pessoais dos participantes se limitarão aos dados necessários para atender aos objetivos do estudo. Os dados serão coletados e processados com as precauções adequadas para garantir a confidencialidade e o sigilo dos participantes. Serão tomadas medidas apropriadas para proteger os dados pessoais contra divulgações ou acessos não autorizados, destruição acidental ou irregular ou perda ou alteração acidental. A confidencialidade dos registros dos participantes será mantida em todos os momentos. Os participantes serão identificados por um número de identificação exclusivo e as informações relativas à sua identidade serão protegidas por meio e acesso restrito ao pesquisador e equipe do estudo. Todas as análises serão realizadas usando dados apropriadamente codificados, sem acesso a informações de identificação pessoal. Todos os relatos do estudo conterão apenas dados agregados e não identificarão participantes individualmente.

6. Resultados Esperados

- Conhecimento da soroprevalência da infecção (prévia) por 15 diferentes tipos de HPV em mulheres imunossuprimidas devido a transplante renal ou hepático ou lúpus eritematoso sistêmico e em mulheres imunocompetentes da mesma faixa etária

- Conhecimento da prevalência da infecção (atual, no momento da inclusão) por 24 diferentes tipos de HPV em mulheres imunossuprimidas devido a transplante renal ou hepático ou lúpus eritematoso sistêmico e em mulheres imunocompetentes da mesma faixa etária

- Avaliação da resposta imune primária e da segurança da vacina de HPV quadrivalente em mulheres imunossuprimidas de 18 a 45 anos. Estudos nesta população são escassos e não há nenhum trabalho semelhante no Brasil. Tais resultados podem dar suporte técnico a decisões do Programa Nacional de Imunizações e do Ministério da Saúde para a ampliação da vacinação de HPV para esta população.

- A avaliação laboratorial (PCR e sorologia) dos tipos de HPV incluídos na vacina HPV9V permitirá avaliar o potencial impacto desta vacina nesta população.

- Este estudo poderá servir de base para estudo posterior sobre persistência de anticorpos antiHPV após imunização com a vacina de HPV quadrivalente nesta população de mulheres imunossuprimidas devido a transplante renal ou hepático ou lúpus eritematoso sistêmico e em mulheres imunocompetentes da mesma faixa etária

7. Desafios científicos e tecnológicos e os meios e métodos para superá-los:

Não há nenhum laboratório no Brasil que realize sorologia de HPV. Testes sorológicos e antígenos de HPV não estão disponíveis comercialmente. Os laboratórios produtores das vacinas de HPV desenvolveram e detêm a tecnologia dos testes sorológicos para detecção e dosagem de anticorpos anti-HPV. Porém, o Dr. Joakim Dillner do Departament of Laboratory Medicine, Karolinska Institute, Suécia, desenvolveu uma metodologia (PsV-Luminex) já validada para detecção de anticorpos de HPV e realizará os testes sorológicos para o presente estudo[38].

8. Formação de recursos humanos

O projeto envolverá a três alunos de pós-graduação (dois doutorados e um mestrado) e dois alunos de iniciação científica.

9. Disseminação e avaliação:

Os resultados deste estudo serão divulgados em congressos científicos e publicados em revistas científicas.

10. Orçamento

Ítem	Valor (R$)
Detecção e genotipagem HPV	62.521,00
Testes sorológicos de HPV	50.920,00
Citologia base líquida	9.750,00
Outros	6.158,29
Transporte material biológico	5.910,72
Total Geral	**135.260,01**

OBS. Orçamento detalhado na página seguinte (pag. 25)

11. Financiamento

O financiamento para este estudo será solicitado à Fundação de Amparo à Pesquisa do Estado de São Paulo (FAPESP).

12. Outros apoios ao estudo:

O Programa Nacional de Imunizações / Ministério da Saúde dará apoio ao estudo por meio do fornecimento de 1.125 doses de vacinas de HPV4V.

13. Orçamento detalhado

	Quantidade (/participante)	Quantidade (total)	Valor unitário	Valor total (R$)
**Material de consumo**				
**1. Detecção e genotipagem de HPV**	1 teste	375		
Extração do DNA		375	30,00	11.250,00
Teste para detecção /genotipagem de HPV (Papillocheck, Greiner One) - kits com 60 testes / kit		7	7.080,00	49.560,00
HotStarTaqPolymerase (1000U) - kit 1000 reações		1	1.711,00	1.711,00
**Subtotal PCR / genotipagem**				**62.521,00**
**2. Sorologia de HPV** (+ 200 testes controle)	2 testes (€13/teste*)	950	53,60	**50.920,00**
**3. Material para coleta de sangue**	2 coletas			
Swab de alcool (alcohol prep pads)	caixas com 200 unidades	5	14,50	72,50
Agulha para coleta a vácuo (23x0,8 mm ou 25x0,7)	caixas com 100 unidades	8	56,00	448,00
Adaptador para agulha de coleta múltipla (canhão)	pacotes com 250 unidades	4	88,00	352,00
Tubos (vacutainer) - tampa amarela para sorologia (10 ml)	caixas com 100 unidades	8	122,10	976,80
Adesivo "blood stop"	caixas com 500 unidades	2	18,00	36,00
Algodão seco	Pacotes com 100 gr	8	4,60	36,80
Luvas	caixas com 100 unidades (50 pares)	16	17,89	286,24
Microtubo Eppendorf 2 ml	pacotes com 1000 frascos	3	42,90	128,70
**Subtotal material coleta de sangue**				**2.337,04**
**4. Citologia base líquida**	1 teste	375	26,00	**9.750,00**
**5. Outros**				
Teste rápido de gravidez	3 testes	1.125	1,25	1.406,25
Termômetros	1	375	5,00	1.875,00
Papel A4 (pacotes 500 folhas)		10	16,00	160,00
Toner para impressora		1	380,00	380,00
**Total material de consumo**				**129.349,29**
**Serviços de terceiros**				
Transporte internacional de material biológico (750-1000 amostras/transporte)	(1 transporte = US$1,600**)	1	5.910,72	5.910,72
**TOTAL GERAL**				**135.260,01**

*1€ = R$4,1231 ; ** 1US$ = R$3,6942 (Banco Central do Brasil, 24/03/2016)

14. Cronograma de realização do estudo*

	**2017**		**2018**		**2019**	
	1° quadrimestre	Maio-Dezembro	1° semestre	2° semestre	1° semestre	2° semestre
Aprovação CEP, Solicitação financiamento						
Inclusão						
Vacinação						
Coleta de dados / amostras clínicas						
Realização dos testes sorológicos e PCR-HPV						
Análise dos dados de segurança da vacina e resposta primária à vacinação, no M7 (1 mês após 3ª dose)						
Relato para publicação						

* O projeto só será iniciado após a aprovação pelo Sistema CEP.

15. Bibliografia

1 BONNEZ, W.; REICHMAN, R. C. Papillomaviruses. In: (Ed.). **Mandell, Douglas and Bennett's Principles and Practice of Infectious Diseases**. 7th: Elsevier, v.2, 2010. cap. 144, p.2035-2049.

2 LOWY, D. R. et al. Primary endpoints for future prophylactic human papillomavirus vaccine trials: towards infection and immunobridging. **Lancet Oncol,** v. 16, n. 5, p. e226-33, May 2015. ISSN 1474-5488. Disponível em: < http://www.ncbi.nlm.nih.gov/pubmed/25943067 >.

3 HERRERO, R.; GONZÁLEZ, P.; MARKOWITZ, L. E. Present status of human papillomavirus vaccine development and implementation. **Lancet Oncol,** v. 16, n. 5, p. e206-16, May 2015. ISSN 1474-5488. Disponível em: < http://www.ncbi.nlm.nih.gov/pubmed/25943065 >.

4 WHO. **Human papillomavirus (HPV) and cervical cancer**. Genebra: World Health Organization 2015.

5 CAPOTE NEGRIN, L. G. Epidemiology of cervical cancer in Latin America. **Ecancermedicalscience,** v. 9, p. 577, 2015. ISSN 1754-6605. Disponível em: < http://www.ncbi.nlm.nih.gov/pubmed/26557875 >.

6 REUSSER, N. M. et al. HPV Carcinomas in Immunocompromised Patients. **J Clin Med,** v. 4, n. 2, p. 260-81, 2015. ISSN 2077-0383. Disponível em: < http://www.ncbi.nlm.nih.gov/pubmed/26239127 >.

7 PALEFSKY, J. M.; GILLISON, M. L.; STRICKLER, H. D. Chapter 16: HPV vaccines in immunocompromised women and men. **Vaccine,** v. 24 Suppl 3, p. S3/140-6, Aug 2006. ISSN 0264-410X. Disponível em: < http://www.ncbi.nlm.nih.gov/pubmed/16950001 >.

8 HINTEN, F. et al. HPV-related (pre)malignancies of the female anogenital tract in renal transplant recipients. **Crit Rev Oncol Hematol,** v. 84, n. 2, p. 161-80, Nov 2012. ISSN 1879-0461. Disponível em: < http://www.ncbi.nlm.nih.gov/pubmed/22425015 >.

9 WIELAND, U.; KREUTER, A.; PFISTER, H. Human papillomavirus and immunosuppression. **Curr Probl Dermatol,** v. 45, p. 154-65, 2014. ISSN 1662-2944. Disponível em: < http://www.ncbi.nlm.nih.gov/pubmed/24643184 >.

10 GORMLEY, R. H.; KOVARIK, C. L. Human papillomavirus-related genital disease in the immunocompromised host: Part I. **J Am Acad Dermatol,** v. 66, n. 6, p. 867.e1-14; quiz 881-2, Jun 2012. ISSN 1097-6787. Disponível em: < http://www.ncbi.nlm.nih.gov/pubmed/22583720 >.

11 WAISBERG, M. G. et al. Human papillomavirus and chlamydia trachomatis infections in rheumatoid arthritis under anti-TNF therapy: an observational study. **Rheumatol Int,** v. 35, n. 3, p. 459-63, Mar 2015. ISSN 1437-160X. Disponível em: < http://www.ncbi.nlm.nih.gov/pubmed/25348220 >.

12 LYRIO, L. D. et al. Prevalence of cervical human papillomavirus infection in women with systemic lupus erythematosus. **Rheumatol Int,** v. 33, n. 2, p. 335-40, Feb 2013. ISSN 1437-160X. Disponível em: < http://www.ncbi.nlm.nih.gov/pubmed/22451033 >.

13 KLUMB, E. M. et al. Are women with lupus at higher risk of HPV infection? **Lupus,** v. 19, n. 13, p. 1485-91, Nov 2010. ISSN 1477-0962. Disponível em: < http://www.ncbi.nlm.nih.gov/pubmed/20605875 >.

14 COSTAPINTO, L. et al. Prevalence of Chlamydia trachomatis endocervical infection in systemic lupus erythematosus patients and evaluation of the risk for HPV-induced lesions. **Rheumatol Int,** v. 33, n. 3, p. 631-6, Mar 2013. ISSN 1437-160X. Disponível em: < http://www.ncbi.nlm.nih.gov/pubmed/22484838 >.

15 JOURA, E. A. et al. A 9-valent HPV vaccine against infection and intraepithelial neoplasia in women. **N Engl J Med,** v. 372, n. 8, p. 711-23, Feb 2015. ISSN 1533-4406. Disponível em: < http://www.ncbi.nlm.nih.gov/pubmed/25693011 >.

16 Human papillomavirus vaccines: WHO position paper, October 2014. **Wkly Epidemiol Rec,** v. 89, n. 43, p. 465-91, Oct 2014. ISSN 0049-8114. Disponível em: < http://www.ncbi.nlm.nih.gov/pubmed/25346960 >.

17 DAY, P. M. et al. In vivo mechanisms of vaccine-induced protection against HPV infection. **Cell Host Microbe,** v. 8, n. 3, p. 260-70, Sep 2010. ISSN 1934-6069. Disponível em: < http://www.ncbi.nlm.nih.gov/pubmed/20833377 >.

18 SCHILLER, J. T.; CASTELLSAGUÉ, X.; GARLAND, S. M. A review of clinical trials of human papillomavirus prophylactic vaccines. **Vaccine,** v. 30 Suppl 5, p. F123-38, Nov 2012. ISSN 1873-2518. Disponível em: < http://www.ncbi.nlm.nih.gov/pubmed/23199956 >.

19 TJALMA, W. A. There are two prophylactic human papillomavirus vaccines against cancer, and they are different. **J Clin Oncol,** v. 33, n. 8, p. 964-5, Mar 2015. ISSN 1527-7755. Disponível em: < http://www.ncbi.nlm.nih.gov/pubmed/25667272 >.

20 LUNA, J. et al. Long-term follow-up observation of the safety, immunogenicity, and effectiveness of Gardasil™ in adult women. **PLoS One,** v. 8, n. 12, p. e83431, 2013. ISSN 1932-6203. Disponível em: < https://www.ncbi.nlm.nih.gov/pubmed/24391768 >.

21 **Esclarecimento sobre o registro das vacinas contra HPV**. BIOLÓGICOS, S. D. M. E. P. e MONITORAMENTO, S. D. C. E. Brasil: Agência Nacional de Vigilância Sanitária 2015.

22 TOFT, L. et al. Vaccination against oncogenic human papillomavirus infection in HIV-infected populations: review of current status and future perspectives. **Sex Health,** v. 11, n. 6, p. 511-23, Dec 2014. ISSN 1448-5028. Disponível em: < http://www.ncbi.nlm.nih.gov/pubmed/25218800 >.

23 GOMEZ-LOBO, V. et al. Immunogenicity of a prophylactic quadrivalent human papillomavirus L1 virus-like particle vaccine in male and female adolescent transplant recipients. **Pediatr Transplant,** v. 18, n. 3, p. 310-5, May 2014. ISSN 1399-3046. Disponível em: < http://www.ncbi.nlm.nih.gov/pubmed/24484551 >.

24 KUMAR, D. et al. Immunogenicity of quadrivalent human papillomavirus vaccine in organ transplant recipients. **Am J Transplant,** v. 13, n. 9, p. 2411-7, Sep 2013. ISSN 1600-6143. Disponível em: < http://www.ncbi.nlm.nih.gov/pubmed/23837399 >.

25 PELLEGRINO, P.; RADICE, S.; CLEMENTI, E. Immunogenicity and safety of the human papillomavirus vaccine in patients with autoimmune diseases: A systematic review. **Vaccine,** v. 33, n. 30, p. 3444-9, Jul 2015. ISSN 1873-2518. Disponível em: < http://www.ncbi.nlm.nih.gov/pubmed/26036945 >.

26 JACOBSON, D. L. et al. Immunogenicity and tolerability to human papillomavirus-like particle vaccine in girls and young women with inflammatory bowel disease. **Inflamm Bowel Dis,** v. 19, n. 7, p. 1441-9, Jun 2013. ISSN 1536-4844. Disponível em: < http://www.ncbi.nlm.nih.gov/pubmed/23567780 >.

27 MOK, C. C. et al. Immunogenicity and safety of a quadrivalent human papillomavirus vaccine in patients with systemic lupus erythematosus: a case-control study. **Ann Rheum Dis,** v. 72, n. 5, p. 659-64, May 2013. ISSN 1468-2060. Disponível em: < http://www.ncbi.nlm.nih.gov/pubmed/22589375 >.

28 SOYBILGIC, A. et al. Safety and immunogenicity of the quadrivalent HPV vaccine in female Systemic Lupus Erythematosus patients aged 12 to 26 years. **Pediatr Rheumatol Online J,** v. 11, p. 29, 2013. ISSN 1546-0096. Disponível em: < http://www.ncbi.nlm.nih.gov/pubmed/23924237 >.

29 HEIJSTEK, M. W. et al. Immunogenicity of the bivalent human papillomavirus vaccine in adolescents with juvenile systemic lupus erythematosus or juvenile dermatomyositis. **J Rheumatol,** v. 40, n. 9, p. 1626-7, Sep 2013. ISSN 0315-162X. Disponível em: < http://www.ncbi.nlm.nih.gov/pubmed/23997002 >.

30 WILKIN, T. et al. Safety and immunogenicity of the quadrivalent human papillomavirus vaccine in HIV-1-infected men. **J Infect Dis,** v. 202, n. 8, p. 1246-53, Oct 2010. ISSN 1537-6613. Disponível em: < http://www.ncbi.nlm.nih.gov/pubmed/20812850 >.

31 GIACOMET, V. et al. Safety and immunogenicity of a quadrivalent human papillomavirus vaccine in HIV-infected and HIV-negative adolescents and young adults. **Vaccine,** v. 32, n. 43, p. 5657-61, Sep 2014. ISSN 1873-2518. Disponível em: < http://www.ncbi.nlm.nih.gov/pubmed/25149430 >.

32 KAHN, J. A. et al. Immunogenicity and safety of the human papillomavirus 6, 11, 16, 18 vaccine in HIV-infected young women. **Clin Infect Dis,** v. 57, n. 5, p. 735-44, Sep 2013. ISSN 1537-6591. Disponível em: < http://www.ncbi.nlm.nih.gov/pubmed/23667266 >.

33 SAÚDE, M. D. et al. **INFORME TÉCNICO SOBRE A VACINA PAPILOMAVÍRUS HUMANO (HPV) NA ATENÇÃO BÁSICA**: Ministério da Saúde 2013.

34 IMUNIZAÇÕES, C. G. D. P. N. D. **Boletim informativo do PNI 2015 - Vacinação contra HPV**. Brazil: Ministério da Saúde 2015.

35 SAÚDE, M. D.; TRANSMISSÍVEIS, D. D. V. D. D.; IMUNIZAÇÃO, C. G. D. P. N. D. **INFORME TÉCNICO DA VACINA PAPILOMAVÍRUS HUMANO 6, 11, 16 E 18 (RECOMBINANTE) 2015**. Brasil: Ministério da Saúde 2015.

[36] **Nota informativa sobre mudanças no calendário nacional de vacinação para o ano de 2017**. PROGRAMA e IMUNIZAÇÕES, N. D. Brasil: Ministério da Saúde 2017.

[37] UČAKAR, V. et al. Pre-vaccination seroprevalence of 15 human papillomavirus (HPV) types among women in the population-based Slovenian cervical screening program. **Vaccine,** v. 31, n. 43, p. 4935-9, Oct 2013. ISSN 1873-2518. Disponível em: < http://www.ncbi.nlm.nih.gov/pubmed/23994822 >.

[38] FAUST, H. et al. Validation of multiplexed human papillomavirus serology using pseudovirions bound to heparin-coated beads. **J Gen Virol,** v. 91, n. Pt 7, p. 1840-8, Jul 2010. ISSN 1465-2099. Disponível em: < http://www.ncbi.nlm.nih.gov/pubmed/20181747 >.

[39] SERVICES, U. S. D. O. H. A. H.; ADMINISTRATION, F. A. D.; RESEARCH, C. F. B. E. A. **Guidance for Industry: Toxicity Grading Scale for Healthy Adult and Adolescent Volunteers Enrolled in Preventive Vaccine Clinical Trials**. USA 2007.

[40] SAÚDE, M. D. **Manual de Vigilância Epidemiológica de Eventos Adversos Pós-Vacinação**. TRANSMISSÍVEIS, D. D. V. D. D. Brazil: Ministério da Saúde**:** 34 p. 2014.

[41] DALSTEIN, V. et al. Analytical evaluation of the PapilloCheck test, a new commercial DNA chip for detection and genotyping of human papillomavirus. **J Virol Methods,** v. 156, n. 1-2, p. 77-83, Mar 2009. ISSN 0166-0934. Disponível em: < http://www.ncbi.nlm.nih.gov/pubmed/19041893 >.

[42] SAÚDE, M. D.; CÂNCER, I. N. D. **Diretrizes Brasileiras para Rastreamento do Câncer de Colo do Útero**. Brazil 2011.

[43] EKLUND, C. et al. International collaborative proficiency study of Human Papillomavirus type 16 serology. **Vaccine,** v. 30, n. 2, p. 294-9, Jan 2012. ISSN 1873-2518. Disponível em: < http://www.ncbi.nlm.nih.gov/pubmed/22079074 >.

[44] GRABOWSKA, K. et al. Evaluation of cost-precision rations of different strategies for ELISA measurement of serum antibody levels. **J Immunol Methods,** v. 271, n. 1-2, p. 1-15, Dec 2002. ISSN 0022-1759. Disponível em: < http://www.ncbi.nlm.nih.gov/pubmed/12445724 >.
