# Supplementary material for: Human papillomavirus (HPV) seroprevalence, cervical HPV prevalence and cervical lesions in systemic lupus erythematosus (SLE) and immunocompetent women
Source: Lupus Sci Med. 2026 Apr 9;13(1):e001812. doi: 10.1136/lupus-2025-001812 (PMC13084834; doi:10.1136/lupus-2025-001812)
Supplement: online supplemental file 2 [file lupus-13-1-s002.pdf]

HOSPITAL DAS CLÍNICAS DA FACULDADE DE MEDICINA DA UNIVERSIDADE DE SÃO PAULO-HCFMUSP
TERMO DE CONSENTIMENTO LIVRE E ESCLARECIDO

DADOS DA PESQUISA

Título da pesquisa – Avaliação da resposta imune primária e segurança da vacina de HPV em mulheres em uso de drogas imunossupressoras devido a transplante de órgãos sólidos (rim ou fígado) ou doença reumatológica (Lúpus Eritematoso Sistêmico)

Pesquisador principal – Ana Marli Christovam Sartori

Departamento/Instituto - Departamento de Moléstias Infecciosas e Parasitárias da Faculdade de Medicina da Universidade de São Paulo (FMUSP)

AVALIAÇÃO DO RISCO DA PESQUISA:

( )RISCO MÍNIMO	( )RISCO MÉDIO
(X)RISCO BAIXO	( )RISCO MAIOR

Convidamos a Sra. para participar desta pesquisa de avaliação da vacina de HPV em mulheres imunodeprimidas.

Justificativa e objetivos do estudo – A infecção por HPV (papilomavírus humano) é muito frequente e regride espontaneamente na maioria das vezes. Alguns tipos de HPV chamados de "alto risco" estão associados ao desenvolvimento de câncer do colo do útero, vagina, vulva, ânus, pênis, orofaringe e boca. A infecção persistente por HPV é a principal causa de câncer de colo de útero. Os HPV chamados de "baixo risco" são causa das verrugas genitais ("crista de galo"). Pessoas imunodeprimidas têm maior risco de desenvolver doenças associadas ao HPV. As principais formas de prevenção do câncer do colo do útero são a realização de exame periódico de Papanicolau, que detecta lesões precursoras de câncer, que poderão ser adequadamente tratadas. Atualmente, são também disponíveis as vacinas de HPV.

Existem duas vacinas de HPV registradas no Brasil: uma vacina bivalente, que contém sorotipos 16 e 18, responsáveis por 70% dos cânceres de colo de útero no país; e uma vacina quadrivalente (HPV4v) que contém, além dos sorotipos 16 e 18, os sorotipos 6 e 11, responsáveis por 90% das verrugas genitais. A vacina quadrivalente de HPV é recomendada na rotina do Programa Nacional de Imunizações para todas as meninas de 9 a 13 anos e meninos de 12 a 13 anos de idade e homens e mulheres vivendo com HIV de 14 a 26 anos. Esta vacina está licenciada no país para uso em meninas e mulheres de 9 anos a 45 anos e homens de 9 anos a 26 anos. Poucos estudos avaliaram a vacina de HPV em mulheres em uso de tratamento imunossupressor devido transplante de órgãos ou doenças reumatológicas. Nesses estudos, a vacina foi bem tolerada, porém foi observada uma diminuição da resposta de anticorpos à vacinação, quando comparadas com mulheres saudáveis. Nenhum desses estudos foi realizado no Brasil.

Você está sendo convidada a participar de uma pesquisa que tem como objetivo avaliar a segurança e a resposta de anticorpos à vacina quadrivalente de HPV em mulheres de 18 a 45 anos, em uso de medicamento imunossupressor e comparar com mulheres sem imunossupressão da mesma faixa etária. Para participar, você

não pode ter recebido nenhuma dose de vacina de HPV anteriormente. Se concordar, deve assinar o Termo de Consentimento Livre e Esclarecido após ler todo este documento e tirar suas dúvidas com os pesquisadores.

Procedimentos que serão realizados e métodos que serão empregados –
Sua participação neste estudo tem duração de sete meses e começa no dia em que você assinar este documento. Você deverá comparecer a 4 visitas durante o estudo (nos Meses 0, 2, 6, 7).

Todas as participantes receberão três doses da vacina quadrivalente de HPV (tempo 0, 2 meses e 6 meses), conforme esquema recomendado para mulheres de 18 a 45 anos.

Todas as participantes deverão responder a um questionário que inclui perguntas sobre sua condição clínica (doenças prévias e atuais, medicamentos em uso).

Dados sobre a sua doença, seus exames e medicamentos serão consultados em seu prontuário médico desta instituição. Essas informações serão utilizadas apenas neste estudo, sendo garantido sigilo dos dados.

Todas as participantes serão submetidas a exame ginecológico com coleta de amostra clínica para realização de teste de Papanicolau e teste de PCR para detecção de HPV antes de iniciar a vacinação (Mês 0). O exame ginecológico e o Papanicolau serão repetidos sete meses após o exame inicial, de acordo com as recomendações do Ministério da Saúde para o acompanhamento de mulheres imunodeprimidas. Mulheres que não iniciaram a vida sexual poderão ser incluídas no estudo, mas não serão submetidas a exame ginecológico e coleta de Papanicolau e teste de detecção de HPV.

Todas as participantes deverão coletar amostras de sangue para pesquisa de anticorpos contra HPV, para avaliar infecção anterior por HPV e a resposta à vacinação. A coleta de sangue será realizada antes de iniciar a vacinação e e um mês após completar a vacinação (nos Meses 0, e 7). Em cada ocasião, serão coletados 10 ml (aproximadamente duas colheres de sopa) de sangue.

Serão também realizados: um teste rápido de HIV, antes de iniciar a vacinação, e teste de gravidez, antes de cada dose da vacina. O exame de HIV será realizado após orientações e aconselhamento e seu resultado será revelado a você pelo médico em até 30 minutos, em ambiente privativo. Caso seja detectado resultado positivo, a candidata não poderá participar do estudo, e receberá orientações e encaminhamento adequados. Em caso de teste de gravidez positivo em qualquer momento do estudo, a participante não receberá as doses subsequentes da vacina e será encaminhada para seguimento.

Após receber cada uma das três doses da vacina de HPV, você deverá permanecer no serviço por um período de 30 minutos, para avaliação de reações imediatas à vacinação. Você receberá também um diário, para anotação de todos os sintomas que apresentar nos primeiros sete dias após cada dose da vacina, e um termômetro, para controle da temperatura nos primeiros sete dias após cada dose da vacina. Entre o terceiro e quinto dia após cada dose da vacina (em geral, no terceiro dia), um dos pesquisadores entrará em contato com você, por telefone, email, SMS ou What'sApp, e fará perguntas sobre sintomas que você poderá apresentar após a vacinação.

Riscos e desconfortos – Os desconfortos e riscos esperados nos procedimentos do estudo relacionam-se: 1. Ao tempo que você gastará vindo às consultas médicas; 2. Ao procedimento de coleta de sangue; 3. Ao

______________________ ______________________
Participante/Representante Pesquisador

procedimento de exame ginecológico, coleta de Papanicolau e teste de PCR para detecção do HPV; 4. Aplicação da vacina quadrivalente de HPV por via intramuscular. Reações no local de aplicação são relatados: dor, vermelhidão e inchaço foram relatados, respectivamente, em até 80%, 25% e 25% dos vacinados, porém são de curta duração e resolvem espontaneamente. Também podem ocorrer cefaleia e desmaio imediatamente após vacinação, atribuída a síndrome vasovagal, que se caracteriza pela diminuição da pressão arterial e do batimento cardíaco por ação do nervo vago. Os primeiros sinais dessa síndrome são: fraqueza, palidez, calor, náusea, tontura, borramento visual e palpitações. Esses sintomas melhoram na posição deitada. Como precaução, pessoas vacinadas deverão permanecer sentadas ou deitadas, em observação, por 30 minutos após a vacinação.

Não há evidências consistentes de associação da vacina com doenças autoimunes, como síndrome de Guillain-Barré, doenças desmielinizantes, tireoidite autoimune ou doenças to tecido conectivo (lúpus, artrite reumatóide, ou artrite juvenil). Nos poucos estudos que avaliaram a vacina em pessoas em uso de imunossupressores, a vacina foi bem tolerada.

O Hospital das Clínicas da FMUSP, onde está situado o CRIE-HCFMUSP, com o apoio do Programa Nacional de Imunizações (portaria nº48 de 28 de julho de 2004), será responsável pela assistência imediata e integral à saúde necessária em caso de dano ou prejuízo decorrente diretamente da vacina de HPV4v utilizada no estudo.

Em caso de dano ou prejuízo decorrente da vacina de HPV4v utilizada no estudo, você terá direito a buscar indenização nos órgãos judiciais competentes.

Benefícios – O participante poderá se beneficiar com a vacinação e a avaliação dos níveis de anticorpos protetores conferidos pela vacinação. Além disso poderá se beneficiar com a realização do teste de PCR para detecção e genotipagem de HPV, que não é realizado na rotina. Não haverá compensação financeira por sua participação. As atividades (exames e consultas) da pesquisa serão gratuitas.

Todas as participantes têm plena liberdade de recusar-se a participar ou retirar o seu consentimento em qualquer fase da pesquisa sem qualquer interferência com o seu tratamento.

Os dados serão coletados e processados com as precauções adequadas para garantir a confidencialidade dos dados e o sigilo das participantes, que serão mantidos em todos os momentos do estudo. As participantes serão identificadas por um número de identificação exclusivo e apenas o pesquisador e equipe do estudo terão acesso às informações relativas à sua identidade. Todas as análises serão realizadas usando dados codificados, sem acesso a informações de identificação pessoal. Todos os relatos do estudo conterão apenas dados agregados e não identificarão participantes individualmente.

Todas as participantes receberão uma via do termo de consentimento.

Em qualquer etapa do estudo, você terá acesso aos profissionais responsáveis pela pesquisa para esclarecimento de dúvidas. A principal investigadora é a Dra. Ana Marli Christovam Sartori, que pode ser encontrado no endereço: Av. Dr. Enéas de Carvalho Aguiar, 155, São Paulo, SP ou pelo telefone (11) 2661-6392. Se você tiver alguma consideração ou dúvida sobre a ética da pesquisa, entre em contato com o Comitê de Ética em Pesquisa (CEP), que é um órgão independente, cuja função é defender os interesses, a integridade e a dignidade dos participantes de projetos de pesquisa – Rua Ovídio Pires de Campos, 225 – 5º andar horário

______________________ ______________________

Participante/Representante Pesquisador

de funcionamento: segunda a sexta, exceto feriados, das 8:00h às 16:00h– tel: (11) 2661-7585, 2661-1548, 2661-1549; e-mail: cappesq.adm@hc.fm.usp.br.

Fui suficientemente informada a respeito do estudo "**Avaliação da resposta primária e segurança da vacina de HPV em mulheres em uso de drogas imunossupressoras devido transplante de órgãos sólidos (rim ou fígado) ou doença reumatológica (Lúpus Eritematoso Sistêmico)**". Eu discuti as informações acima com o Pesquisador Responsável (..) ou pessoa (s) por ele delegada (s) (..) sobre a minha decisão em participar nesse estudo. Ficaram claros para mim os objetivos, os procedimentos, os potenciais desconfortos e riscos e as garantias. Concordo voluntariamente em participar deste estudo, assino este termo de consentimento e recebo uma via rubricada pelo pesquisador.

____	___________________________________
Assinatura do participante /representante legal	Assinatura do responsável pelo estudo
Data: / /	Data: / /

Durante o desenvolvimento desta pesquisa, o material biológico (sangue) será armazenado Laboratório de Virologia do IMT-FMUSP, sob responsabilidade de José Eduardo Levi (telefone: (11)3061-7011) de acordo com a resolução 441/2011 e poderá ser utilizado para análises relativas a este projeto de pesquisa.

Será solicitada assinatura de novo TCLE para o armazenamento das amostras que sobrarem após a realização dos exames desta pesquisa, que poderão ser guardadas no Biobanco do IMT-FMUSP. Se você não permitir o armazenamento, sua amostra será destruída após o término deste estudo. O tempo em que seu material ficará armazenado será autorizado pela Comissão de Ética do HCFMUSP. A sra. pode retirar esta permissão a qualquer momento.

DADOS DE IDENTIFICAÇÃO DO PARTICIPANTE DA PESQUISA OU RESPONSÁVEL LEGAL

NOME:...

DOCUMENTO DE IDENTIDADE Nº : .... SEXO : .M □ F □

DATA NASCIMENTO: ..../......../...... ENDEREÇO .... Nº ....

APTO:..................BAIRRO: ....CIDADE ....

CEP:...................................... TELEFONE: DDD (............) ....

RESPONSÁVEL LEGAL ....

NATUREZA (grau de parentesco, tutor, curador etc.) ....

DOCUMENTO DE IDENTIDADE:...................................SEXO: M □ F □

DATA DE NASCIMENTO.: ....../......./......

ENDEREÇO: .... Nº .... APTO: .... BAIRRO: .... CIDADE: .... CEP: ....

TELEFONE: DDD (............)..

______________________	______________________
Participante/Representante	Pesquisador
