# Supplementary material for: Human papillomavirus (HPV) seroprevalence, cervical HPV prevalence and cervical lesions in systemic lupus erythematosus (SLE) and immunocompetent women
Source: Lupus Sci Med. 2026 Apr 9;13(1):e001812. doi: 10.1136/lupus-2025-001812 (PMC13084834; doi:10.1136/lupus-2025-001812)
Supplement: online supplemental file 3 [file lupus-13-1-s003.docx]

**SUPPLEMENTARY FILE**

Supplementary Table 1: **Association** **of systemic lupus erythematosus (SLE) and cervical cytological lesions (HSIL or LSIL vs. benign cellular changes) (n=202)**

| **Variable** | **Adjusted OR*** | **95% CI** | **p-value** |
| --- | --- | --- | --- |
| Immunocompetent | 1.00 | Reference | – |
| SLE | **6.62** | **1.65 – 26.66** | **0.008** |
| **Age at inclusion (years)** | 0.95 | 0.88 – 1.03 | 0.223 |
| **Educational level (Years)** | 0.98 | 0.83 – 1.15 | 0.787 |
| **Number of sexual partners** | 1.04 | 0.93 – 1.16 | 0.484 |

The analysis included women with SLE (n = 91) and immunocompetent controls (n = 111).
Odds ratios were estimated using logistic regression adjusted for age, educational level and number of sexual partners.

HSIL = high-grade squamous intraepithelial lesion; LSIL = low-grade squamous intraepithelial lesion

Supplementary Table 2: **Association of lupus treatment with or without mycophenolate mofetil and cervical lesions (HSIL or LSIL vs. benign cellular changes)** **among women with systemic lupus erythematosus (n = 90)**

| **Variable** | **Adjusted OR** | **95% CI** | **p-value** |
| --- | --- | --- | --- |
| **Mycophenolate mofetil use (yes vs no)** | **5.08** | **1.38 – 18.79** | **0.015** |
| Age (years) | 0.93 | 0.84 – 1.02 | 0.125 |
| Educational level (years) | 1.04 | 0.86 – 1.27 | 0.690 |
| Number of sexual partners | 1.03 | 0.90 – 1.18 | 0.647 |

Odds ratios were estimated using logistic regression restricted to women with systemic lupus erythematosus.

HSIL = high-grade squamous intraepithelial lesion; LSIL = low-grade squamous intraepithelial lesion

Supplementary Table 3: **Association of disease activity score (SLEDAI*) and HPV-related outcomes among women with systemic lupus erythematosus**

| **Outcome** | **n** | **Adjusted OR** | **95% CI** | **p-value** |
| --- | --- | --- | --- | --- |
| HPV seropositivity (any type**) | 104 | 1.09 | 0.47 – 2.55 | 0.84 |
| Cervical HPV DNA positivity (any type***) | 93 | 1.22 | 0.61 – 2.44 | 0.57 |
| Cervical lesion (LSIL or HSIL) vs normal cytology | 91 | 1.13 | 0.42 – 3.05 | 0.81 |

Odds ratios were estimated using logistic regression adjusted for age at inclusion, educational level and number of sexual partners.

* SLEDAI ≤4 = Remission/low disease activity or >4 = Active disease

** HPV types tested (serology): 6, 11, 16, 18, 31, 33, 45, 52, and 58

*** HPV types tested (PCR): 6, 11, 16, 18, 31, 33, 35, 39, 40, 41, 42, 43, 44/55, 45, 51, 52, 53, 56, 58, 59, 66, 68, 70, 73 and 82

Due to the limited number of events and evidence of data separation in some models, these analyses should be interpreted as exploratory.

Supplementary Table 4: **Association of systemic lupus erythematosus (SLE) and** **cervical HPV DNA positivity (any type*)**

| **Variable** | **Adjusted OR** | **95% CI** | **p-value** |
| --- | --- | --- | --- |
| Immunocompetent | 1.00 | Reference | – |
| **SLE** | **2.36** | **1.17 – 4.77** | **0.017** |
| **Age at inclusion (years)** | 0.96 | 0.91 – 1.00 | 0.060 |
| **Educational level (years)** | 0.95 | 0.86 – 1.05 | 0.302 |
| **Number of sexual partners** | 1.01 | 0.94 – 1.08 | 0.832 |

Odds ratios were estimated using logistic regression adjusted for age, educational level and number of sexual partners. SLE: n = 93; immunocompetent: n = 114

* HPV types tested (PCR): 6, 11, 16, 18, 31, 33, 35, 39, 40, 41, 42, 43, 44/55, 45, 51, 52, 53, 56, 58, 59, 66, 68, 70, 73 and 82

Supplementary Table 5: **Association of systemic lupus erythematosus (SLE) and HPV seropositivity (any type*) (n = 223)**

| **Variable** | **Adjusted OR** | **95% CI** | **p-value** |
| --- | --- | --- | --- |
| Immunocompetent | 1.00 | Reference | – |
| SLE | **2.58** | **1.28 – 5.20** | **0.008** |
| **Age at inclusion (years)** | 1.03 | 0.98 – 1.08 | 0.224 |
| **Educational level (years)** | 0.91 | 0.82 – 1.01 | 0.087 |
| **Number of sexual partners*** | **1.21** | **1.06 – 1.37** | **0.003** |

Odds ratios were estimated using logistic regression adjusted for: age, educational level and number of sexual partners. SLE: n = 104; immunocompetent: n = 119

*HPV types tested (PCR): 6, 11, 16, 18, 31, 33, 35, 39, 40, 41, 42, 43, 44/55, 45, 51, 52, 53, 56, 58, 59, 66, 68, 70, 73 and 82
